# Systematic evaluation of the impact of ChIP-seq read designs on genome coverage, peak identification, and allele-specific binding detection

**DOI:** 10.1186/s12859-016-0957-1

**Published:** 2016-02-24

**Authors:** Qi Zhang, Xin Zeng, Sam Younkin, Trupti Kawli, Michael P. Snyder, Sündüz Keleş

**Affiliations:** Department of Statistics, University of Nebraska Lincoln, Lincoln, Nebraska USA; Department of Statistics, University of Wisconsin Madison, Madison, Wisconsin USA; Department of Biostatistics and Medical Informatics, University of Wisconsin Madison, Madison, Wisconsin USA; Department of Genetics, Stanford University School of Medicine, Palo Alto, California USA; Stanford Center for Genomics and Personalized Medicine, Palo Alto, California USA

**Keywords:** ChIP-Seq, Paired-end, Alignment, Peak identification, Allele-specific binding

## Abstract

**Background:**

Chromatin immunoprecipitation followed by sequencing (ChIP-seq) experiments revolutionized genome-wide profiling of transcription factors and histone modifications. Although maturing sequencing technologies allow these experiments to be carried out with short (36–50 bps), long (75–100 bps), single-end, or paired-end reads, the impact of these read parameters on the downstream data analysis are not well understood. In this paper, we evaluate the effects of different read parameters on genome sequence alignment, coverage of different classes of genomic features, peak identification, and allele-specific binding detection.

**Results:**

We generated 101 bps paired-end ChIP-seq data for many transcription factors from human GM12878 and MCF7 cell lines. Systematic evaluations using *in silico* variations of these data as well as fully simulated data, revealed complex interplay between the sequencing parameters and analysis tools, and indicated clear advantages of paired-end designs in several aspects such as alignment accuracy, peak resolution, and most notably, allele-specific binding detection.

**Conclusions:**

Our work elucidates the effect of design on the downstream analysis and provides insights to investigators in deciding sequencing parameters in ChIP-seq experiments. We present the first systematic evaluation of the impact of ChIP-seq designs on allele-specific binding detection and highlights the power of pair-end designs in such studies.

**Electronic supplementary material:**

The online version of this article (doi:10.1186/s12859-016-0957-1) contains supplementary material, which is available to authorized users.

## Background

Chromatin immunoprecipitation followed by high-throughput sequencing (ChIP-seq) is widely used for genome-wide profiling of histone modifications [1] and transcription factor (TF)-DNA interactions [2, 3]. Popular applications of ChIP-seq include identifying binding sites of a TF in one or more samples [4, 5], comparing histone modifications across two or more samples, and detecting binding differences between alternative alleles (allele-specific binding) when SNP data is available [6–9]. ChIP-seq experiments start with shearing DNA cross-linked with the protein of interest into short fragments. Then, fragments associated with the protein or modification of interest are enriched by immunopreciptitation using an antibody specific to the protein or modification of interest. After purification and size selection, the remaining fragments are sequenced. Currently, the most widely used sequencing platform for ChIP-seq experiments is Illumina. This platform offers many options for experimental design; however, the impact of these design parameters are not well understood. The two key design parameters are read length and read type, i.e., single-end (SE) in which only one end of the fragments are sequenced or paired-end (PE) whereby both ends are sequenced. Intuitively, long PE reads should capture more information than the short SE reads; however, they can cost up to 1.5–2 times more (e.g., one lane of 100 bps PE sequencing costs about 67 % more than one lane of 50 bps SE sequencing at the Biotechnology Center at UW Madison at the time this paper was submitted [10]. Thus, understanding design differences is critical for the cost-effectiveness of genomic research. More importantly, since different designs may be powered differently for various types of discoveries, a comprehensive understanding of design implications is crucial for comparing and integrating ChIP-seq data with different designs.

Recent works on the design of ChIP-seq experiments did not adequately address the performance of PE and SE designs and long and short reads [11–15]. Instead, these studies discussed systematic biases in data generation, sequencing depths of the control and ChIP samples, redundancy of reads, and the impacts of downstream data analysis algorithms. For *Drosophila melanogaster*, they also investigated a limited comparison of PE and SE designs with the exact same read lengths in terms of the library complexity and coverage in repetitive regions using very high depth samples. The human genome has much more repetitive DNA and a greater genome size, and although the sequencing depths of human samples have also been increasing over time, they are still lagging far behind the *Drosophila melanogaster* study. Therefore, it remains largely unclear how the PE and SE designs and long and short reads influence the alignment rates and accuracy, coverage of various repetitive elements, sensitivity and specificity in peak calling and in allele-specific binding detection.

In this paper, we systematically and quantitatively investigated the impact of ChIP-seq read parameters on the alignment, peak identification, and allele-specific binding detection. We first generated PE ChIP-seq data for CTCF, BHLHE40 (also called DEC1), and NONO from the human GM12878 cell line and MAFK from the human MCF7 cell line, as well as the control Input data from these two cell lines, with a read-length of 101 bps at typical depths (15–80 million reads per replicate). We generated data with other read parameters *in silico* from these full data, and evaluated short (36 and 50 bps) and long (75 and 101 bps) PE and SE read designs for their impact on alignment, peak calling, and allele-specific binding (ASB) detection. We complemented these comparisons with evaluations on simulated data where the underlying truth was known and established advantages and disadvantages of different designs in terms of accuracy and power. Our study deepens the understanding on the impact of design in transcription factor ChIP-Seq experiments, and is likely to provide insights on other types of ChIP-Seq experiments.

## Methods

### ChIP-seq data

We generated ChIP-seq datasets for CTCF, NONO, and BHLHE40 (DEC1) in GM12878 cells and MAFK in MCF7 cells as part of the phase 3 of the ENCODE project (released at the ENCODE portal [16] in 2014). The information on the antibodies used for ChIP is available at the ENCODE portal and can be accessed using the following accession numbers CTCF (ENCAB000AXU), BHLHE40 (ENCAB000AEK), NONO (ENCAB134GSH) and MAFK (ENCAB000AIJ). A detailed protocol for the ChIP-seq can also be downloaded from the ENCODE portal [17]. Among these factors, CTCF, BHLHE40, and MAFK are sequence specific transcription factors with known motifs while NONO does not have a well-defined motif. These data sets were chosen based on the availability within the ENCODE community at the time of the research and their ENCODE quality measures [18]. In particular, we excluded data with severe bottlenecking in library complexity [19]. Due to our interests in motif analysis and allele-specific binding, we largely focused on sequence-specific transcription factors, and the cell line with the most complete diploid sequences available at the time of the research (GM12878), but also included MCF7 as a second cell line. We used CTCF, MAFK, and NONO in read alignment comparisons, CTCF, MAFK, and BHLHE40 in peak detection comparisons, and CTCF and BHLHE40 datasets in the ASB detection comparisons. Additional file 1: Table S1 provides the numbers of fragments for each dataset.

### *In silico* generation of ChIP-seq data of other designs from the original data

We randomly sampled one end from each paired-end read to generate single-end reads. We used HOMER software [20] to trim the original reads to 75, 50, and 36 bps for generating designs with shorter read lengths. Additional file 1: Table S2 provides the number of fragments, reads, and sequenced base-pairs in each design.

### Alignments by Bowtie and BWA

We initially compared the alignment results of both Bowtie -v mode [21] and BWA [22]. Bowtie can be set to report only uniquely mapped reads (uni-reads), whereas BWA also reports reads that can be mapped to multiple locations (multi-reads). Our simulation results show that Bowtie and BWA have almost identical coverage and accuracy when their alignment rules are comparable and if the multi-reads in BWA output are filtered. However, if the multi-reads are kept, the alignment accuracy of BWA could be low (Additional file 1: Tables S15–S16). Thus we resorted to using only Bowtie alignments for other comparisons. There are many other different alignment tools, such as Bowtie2 [23] and GEM [24]. However, since alignments with BWA and Bowtie dominate the ChIP-seq applications, we focused our attention to these two aligners. In our study, we used Bowtie with the command “Bowtie -v *𝜗* -I 100 -X 700 -a –best –strata -m 1”, where *𝜗*=0, 1, 2, 3 and “-I 100 -X 700" only applies to PE. Here, *𝜗* denotes the number of mismatches allowed in each read, and 100 and 700 the allowed range of the fragment lengths in PE alignments. These upper and lower bounds were chosen based on BWA estimates of the fragment length from the CTCF dataset to make this parameter comparable between the two aligners. We considered four different options for BWA: “BWA -q 0 -o 0 -n 0.04", “BWA -q 20 -o 0 -n 0.04", “BWA -q 20 -o 1 -n 0.04", and “BWA -q 20 -o 1 -n 8", where -q 0, -o 1 and -n 0.04 are default options of BWA. The option -q allows trimming the low quality base-pairs from their 3^′^ ends, option -o allows user-specified numbers of gaps in the alignment, and option -n controls the number of allowed mismatches. Specifically, when -n is an integer, it denotes the number of allowed mismatches. When -n is between 0 and 1, the number of allowed mismatches is set to the quantile of a Poisson distribution with mean 0.02 ×read-length (after trimming by option -q) with tail probability -n. We used “BWA -q 20 -o 1 -n 8" to generate a relaxed alignment set. After the BWA alignment of the PE designs, we considered keeping all aligned pairs with both ends uniquely mapping (Uni) and additionally kept those with one uniquely mapping end (UR). For SE designs, we kept all the reads that are uniquely mapped (Uni/UR).

### Abbreviations of designs and the design-alignment combinations

For simplicity, we introduced some abbreviations for the designs and the design-alignment combinations in Additional file 1: Tables S3–S7. Some illustrative examples of these abbreviations are as follows. We denoted PE data with read-length 36 bps as PE36, SE data with read-length 75 bps as SE75; the Bowtie alignments in -v mode allowing one mismatch as BOWTIEv1, BOWTIEv1 alignment on PE36 data as PE36v1, and the BOWTIEv2 on SE75 as SE75v2. We used BWAq20o1UR to denote the alignment strategy where we first ran BWA -q 20 -o 1 -n 0.04 and only kept reads that uniquely mapped to the reference genome (SE). For PE, we kept the read pairs with at least one uniquely mapping end.

### ENCODE uniform ChIP-seq processing pipeline

We utilized the uniform ChIP-seq processing pipeline developed and benchmarked by ENCODE [18]. This pipeline utilizes SPP [2] for calling peaks and generates a relaxed peak list with both true peaks and some regions with little or no enrichment of the ChIP-seq signal (3 ×10^5^ by default). Then the irreducible discovery rate (IDR) [25] is applied to threshold the relaxed peak list and generate an optimal peak set for a given IDR level (default 0.02). IDR also accounts for the randomness between replicates. Calculation of the average fragment length is also provided within this pipeline.

### Coverage analysis

We used SPP to estimate the average fragment length for SE designs, and extended the SE reads to this length as their full length. The two ends of the PE reads were connected to form full length fragments.

### Coverage of repetitive elements

We calculated the coverage for each type of repeat element by summing over the fragment lengths of all reads overlapping with the instances of the repeat element and then normalizing the resulting sum with the total length of this type of repetitive element. For both the alignments and the peaks, we only counted those with at least 30 % overlap with repeat elements.

### Measuring alignment accuracy of ChIP-seq data using the relaxed alignment set

We used BWAq20o1n8 results of PE101 as a “gold" standard relaxed set for benchmarking alignment accuracy in real ChIP-seq data analysis where the true origins of the reads were not available. This alignment rule is very relaxed and the alignment locations under this rule are not necessarily accurate. However, if a read cannot be mapped by this rule, it probably should not be mapped by any. Thus, we marked those reads that cannot be aligned in this setting as “unmappable". If an unmappable read was mapped under another design and using another alignment rule, we labeled it as a “false positive" alignment. We then compared the false positive rates in the set differences of the alignments by the two designs. Details of these calculations are illustrated in Additional file 1: Figure S1, e.g., the false positive alignment rate of Design-Alignment 1 is *A*/(*A*+*B*) when it is compared with Design-Alignment 2. In our simulation study, we found that BWAq20o1n8 results of PE101 has very high coverage (median in 5 replicates is 99.15 %), and accuracy (median in 5 replicates is 99.84 %). Even though the simulation settings can never be as complex as the real data, the above results give us confidence about the sensitivity and specificity of BWAq20o1n8 alignment on PE101.

### Motif analysis

For CTCF and MAFK, we used ENCODE defined motifs and corresponding positions weight matrices (PWMs) from [26]. Transcription factor BHLHE40 was not profiled in GM12878 previously and did not have a PWM; therefore, we estimated its PWM from the top 500 peaks of the analysis of PE101v3 data using MEME [27]. The peaks were scanned with these position weight matrices using FIMO [28] for all the motif analysis.

### ChIP-seq peak calling by MOSAiCS + IDR pipeline

Mosaics [5] is a model-based approach for the analysis of ChIP-seq data. We combined it with IDR to get a set of optimal peaks. The pipeline parameters were set as follows: thres: 99th percentile of the bin-level ChIP read count distribution; FDR: 1.0 for generating the set of relaxed peaks. The relaxed peak set was filtered so that the ChIP read counts at the summit were at least as large as the sequencing depth normalized input read counts and the IDR optimal peak set was filtered to contain peaks with at least 10 read counts at the summit.

### Minimax summit distance of the identified peaks

We define minimax summit distance *L*_*M*_(*j*) to measure the spatial distance of the rank *j* peak among the top *M* peaks of the peak lists in the worst-case scenario across all replicates (Additional file 1: Figure S2). Small *L*_*M*_(*j*) indicates better reproducibility. If *L*_*M*_(*j*) is smaller than a given threshold *T*, we label rank *j* peak as reproducible in the top *M* peaks lists. Let *R*_*M*_ be the proportion of reproducible peaks among the top M peaks. Large *R*_*M*_ indicates that the top *M* peaks are more reproducible.

Formally, for a fixed design, let *P*_*ij*_ be the rank *j* peak identified in simulation replicate *i*, where *i*=1,2,3,4,5 and *j*=1,2,…,*M*, and *S*_*ij*_ denote its summit location. The minimax summit distance for rank *j* peaks under the given design is defined as

$$L_{M}(j)\equiv \max_{i,k,i\neq k}\left\{\min_{\ell\leq 1.5M}|S_{ij}-S_{\textit{k}\ell}|\right\}. $$

In the analysis presented in this paper, rank *j* peaks were labeled as reproducible if *L*_*M*_(*j*)≤200 bps, and the overall reproducibility of the top *M* peaks was measured by *R*_*M*_, the proportion of reproducible peaks. We varied *M*∈500×{1,2,…,10}. The exact interpretation of *R*_*M*_ is as follows: if we view two peaks with summit-to-summit distance less than 200 bps as the estimates of the same peak, then for any simulation replicate, the proportion of the targets of its top *M* peaks that can be recovered by the top 1.5*M* peaks from another simulation replicate is at least *R*_*M*_.

### Analysis of peak set differences

When we compared the ranks of the peaks, if the summit distance between two peaks from two peak lists was less than 200 bps, we considered them as two estimates of the same binding event. We compared rankings of the two estimates of the same binding event from different designs in their respective relaxed peak lists. In the coverage comparisons of peaks, we only considered the peaks whose surrounding ± 500 bps windows contained no other peak summits from the other peak list, so that the closely-spaced binding events for which the coverage comparisons were difficult were excluded. Then we defined the coverage around a summit as the number of reads overlapping with its surrounding ± 100 bps window.

### Allele specific binding detection by a modified AlleleSeq pipeline

AlleleSeq [8] employs a binomial test of proportions for allelic imbalance detection. It aligns reads to the paternal and the maternal sequences of the sample to be analyzed and assigns each read to one allele based on the number of mis-matches with the ties broken by random assignment. During this process, it discards the reads with ambiguous base **N**, and those that align to both alleles but at different locations. It then applies the binomial test at the phased heterozygous SNPs with at least 5 reads to test the null hypothesis of no allelic imbalance and achieves FDR control via simulation. In our actual data analysis, we set FDR level to 0.1. AlleleSeq was originally designed for SE reads. We adapted it to the PE setting with the following modification. After the reads were aligned to both alleles by Bowtie, we calculated the number of mis-matches of each fragment by summing the mis-matches from both ends, and then assigned the fragment to one allele based on this total number of mis-matches. We also discarded the reads that were aligned to both alleles with equal number of mis-matches. In both cases, we ran AlleleSeq without a specific peak set, and then overlapped the output with the optimal peak set of PE101v3 for detecting ASB events. AlleleSeq aligns the reads using Bowtie in v mode. We allowed one mismatch for the designs with read length 36 or 50 bps, and two mismatches for those with read lengths 75 or 101 bps.

### ROC curve in ASB detection

In our simulation study, we ranged the cutoff of *p*-value from 0 to 1, and calculated the empirical FDR and true positive rates. Since AlleleSeq excluded the loci with insufficient coverage (<5 reads), the true positive rate did not go to 1, even if we increased the *p*-value cutoff to 1.

### Allele-Specific Open Chromatin (ASOC) regions and Allele-Specific Co-Binding (ASCB)

We pooled the two replicates of 36mer SE DNase-seq data from GM12878 cells from the UCSC ENCODE portal [16] and ran AlleleSeq to detect allele-specific activity. To get a high quality set of ASOC regions, we applied more restrictive rules in filtering and identifying allele-specific behavior. We discarded the SNPs covered by less than 20 reads, and only reported those with *p*-values ≤0.01 and at least 1.5 fold-change between the coverage of two alleles.

In the ASCB analysis of CTCF and BHLHE40, we first defined co-binding events as the events with the summit-to-summit distance of a CTCF peak and a BHLHE40 peak less than 200 bps. Then, the co-binding regions were defined as the union of the ±100 bps windows around these summits. If two or more co-binding regions overlapped, they were merged into a single region. We examined the consistency in allele-specific activity in each of such co-binding regions. We only considered SNPs that were covered by at least 20 reads, had a with *p*-value ≤0.1, and the read proportion from the winning allele were at least 0.7. Within each co-binding region, there could be many SNPs with ASB for one or both TFs. We labeled a co-binding region to be Allele-Specific Co-Binding (ASCB) if both TFs showed ASB in favor of the same allele, Bi-Allele-Specific Binding (BiASB) if the two TFs showed ASB in favor of different alleles. In rare cases, one TF showed ASB in favor of different alleles at different SNPs within the same co-binding region. These cases did not affect the overall conclusions and were excluded.

### Simulation for paired-end reads from the diploid sequence of GM12878 chr19

We simulated paired-end reads from the diploid sequences of chr19 in GM12878 cells using the following procedure.

1. Simulate fragment length *L*: we simulated the fragment lengths by rounding random samples from a shifted beta distribution 100+(700−100)×*Beta*(2,5). This distribution is similar to the empirical distribution of the fragment lengths estimated from BWA paired-end alignments.
2. Simulate the middle point positions of the fragment on the reference sequence *z*: We utilized a read model similar to the model underlying CSEM [29] and cnvCSEM [30]. Specifically, we simulated the middle point positions of the fragments from a discrete distribution where the probability is proportional to the read depth of the pooled CTCF (or Input) data under PE101v3.
3. Simulate the allele assignment *a*∈{*mat, pat*}: We drew a random sample from *Unif*[0.1,0.9] as the true maternal probability in each 1000bps window around SNPs. We further assumed equal maternal probabilities in overlapping windows and maternal probability of 0.5 in regions outside of these windows. We sampled *a* based on the maternal probability of the sampled middle point position.
4. Convert *z* to the corresponding coordinate on the assigned allele *z*^*a*^: We converted *z* to the corresponding coordinate on the assigned allele by the *liftOver* function in the Bioconductor package *rtracklayer*. For the positions on the reference sequence that cannot be mapped to the assigned allele, we slightly perturbed *z* iteratively until it was properly mapped.
5. Extract the read sequences at both ends for the sampled fragment from the assigned allele: We calculated the two end positions of the fragment based on *z*^*a*^ and *L* and extracted the true read sequences from both ends.
6. Sample quality score: For each read, we sampled the quality score from the qualities of the CTCF data.
7. Insert read errors based on the quality score: We calculated the base-wise error probability based on the sampled quality score, determined the read error locations based on the error probabilities, and inserted the read errors based on the error distribution provided in Additional file 1: Table S8.

We performed simulation experiments for five times. Each experimental replicate contains one ChIP sample and one Input sample. The read-densities on the reference genome were from PE101v3 of pooled CTCF and Input samples of GM12878. The ChIP samples contain 2 ×10^6^ reads and the Input sample contains 1.1 ×10^6^ reads, so that the coverage of the simulated samples were similar to the coverage of chr19 in the real data.

In our simulation, the reads were simulated from the diploid sequence. When assessing the accuracy in their alignment to the reference sequence, we treated a read as aligned correctly as long as the extended aligned read covered the true middle point position on the reference sequence. When assessing the accuracy of ASB detection, we defined the true ASB as the SNPs where the ratio between the maternal and the paternal alleles is larger than 1.5 (or smaller than 2/3).

## Results and discussion

The first step of ChIP-seq data analysis is aligning reads to a reference genome, and the choice of alignment strategy impacts the downstream analysis. Thus we started with a detailed comparison of data alignments with different read parameters, and throughout this paper, we paid attention to matching designs and analysis protocols so that the read error rates, the numbers of reads, and the numbers of sequenced bases were comparable when necessary. For simplicity, we introduced some abbreviations for the designs and the design-alignment combinations in Additional file 1: Tables S3–S7 (See also the corresponding section in “Methods”). We also summarized the major comparisons of the design and alignment combinations and the rationale for these comparisons in Table 1. For example, we compared PE36v1 with SE75v2 and also PE50v1 with SE101v2 for a fair comparison of PE and SE designs with similar numbers of sequenced bases and read error rate in alignment. PE designs interrogate the same number of ChIPed fragments with twice as many reads compared to the SE designs. We controlled the numbers of reads by randomly sampling half of the pairs from PE designs, and denoted such designs as PEhalf (Additional file 1: Table S7). At fixed read-lengths, PEhalf designs have the same number of reads and the number of sequenced bases as their SE counterparts, but they could potentially contain less biological information since the paired reads are representing information from the same DNA fragment.

**Table 1:** Descriptions of the design and alignment combinations that are compared throughout the paper

Aim	Comparisons
Compare PE and SE designs with the same number of fragments, similar numbers of sequenced bases, and read error rates	PE36v1 vs SE75v2 and PE50v1 vs SE101v2
Compare PE and SE designs with the same numbers of reads, sequenced bases, and read error rates	PE36v1half vs SE36v1
Compare PE and SE designs with the same number of fragments, read-lengths, and read error rates	PE36v1 vs SE36v1, PE50v1 vs SE50v1, PE75v2 vs SE75v2 and PE101v2 vs SE101v2
Compare long and short PE reads with the same number of fragments and similar read error rates	PE36v1 vs PE75v2 and PE50v1 vs PE101v2
Compare long and short SE reads with the same number of fragments and similar read error rates	SE36v1 vs SE75v2 and SE50v1 vs SE101v2

### The effect of read parameters on alignment coverage and accuracy

#### Alignment rates of Bowtie

Alignment rates have a profound effect on the downstream analysis because higher alignment rates lead to more aligned reads at fixed sequencing depth and typically yield identification of more true signals with higher confidence. Our comparison in alignment rates revealed a complex interplay between the read parameters, alignment rules, and other biological factors (Table 2 and Additional file 1: Tables S9–S14. The alignment of PE requires both ends of each pair to be mapped. This is more stringent than its SE counterpart, but also improves the uniqueness in alignment. As a result, SE designs with longer reads (50, 75, and 101 bps) usually had higher alignment rates than the PE designs with the same read-length, but PE36 had higher alignment rates than SE36 in most cases due to its higher uniqueness in alignment. For example, the alignment rates of SE75v2 were 2–7 % higher than PE75v2, but those of SE36v1 were 1–7.5 % lower than PE36v1 for most data except one replicate of CTCF. The effect of read length is more complicated. Longer SE reads had higher alignment rates than short SE reads in most cases due to their higher sequence uniqueness. In contrast, median read lengths seemed to improve alignment rates for PE, and in most cases, PE50v1 and PE75v2 outperformed PE101v2 and PE36v1 in alignment rates, respectively. The observed lower alignment rates of PE101v2 may be largely due to the lower quality near the 3 ^′^ end of long reads, a phenomenon commonly observed and well characterized for Illumina platforms [31]. However, how much lower it could be depends on the specific quality profile. When the number of sequenced bases was also controlled (e.g., PE36v1 vs. SE75v2), SE designs had 0–5 % higher alignment rates than their PE counterparts. In fact, for all datasets and all the Bowtie alignment settings considered, we found that (1) SE75v3 yielded the highest alignment rates for all except one replicate of CTCF where it was 0.05 % less than SE50v3; and (2) PE50v3 yielded the highest alignment rates in PE for CTCF and BHLHE40, and the second highest in PE for MAFK, where it was 0.11 % and 0.04 % less than PE75v3. The results on the simulated CTCF data showed similar patterns (Additional file 1: Table S15).

**Table 2:** Percentages of aligned reads for replicate 1 of MAFK data

Design	v0	v1	v2	v3	q20o1Uni	q20o1UR	q20o1
SE36	76.76	81.95	82.68	83.00	82.90	82.9	97.49
SE50	78.39	85.30	86.37	86.84	87.24	87.24	96.90
SE75	77.46	86.82	88.36	88.96	90.34	90.34	95.95
SE101	74.45	85.86	87.96	88.72	91.20	91.20	95.35
PE36	75.27	84.40	84.68	84.40	75.45	88.79	95.84
PE50	73.41	85.48	86.56	86.74	81.87	91.21	95.91
PE75	68.39	84.19	86.32	86.85	86.80	92.75	95.77
PE101	63.05	81.55	84.76	85.66	88.28	93.12	95.45

#### Effective genome coverage of Bowtie alignments

One of the key differences between PE and SE designs is that once the PE reads are mapped, genomic coverage, i.e., the numbers of bases spanned by the aligning reads, are readily available whereas this quantity relies on the fragment length estimation in SE designs. We evaluated how the designs differed in coverage, and found that it heavily depended on data quality. In detail, we compared the designs in terms of multi-coverage that we defined as the sizes of the genomic regions (number of bases) covered by at least five reads (Additional file 1: Figure S3).

We used the peak finder SPP [2] to estimate fragment sizes for the SE designs. We observed that PE designs had higher multi-coverage than the comparable SE designs for MAFK and NONO, but not for CTCF. We conjectured that such disagreement is likely due to the antibody quality. We concluded that all the designs might have similar coverage for data with good antibody such as CTCF. However, for typical TFs, PE designs do provide better coverage. We did not find apparent pattern of differences between different read lengths for either the PE or the SE design.

#### Repetitive element coverage of Bowtie alignments of different designs

**Fig. 1:**
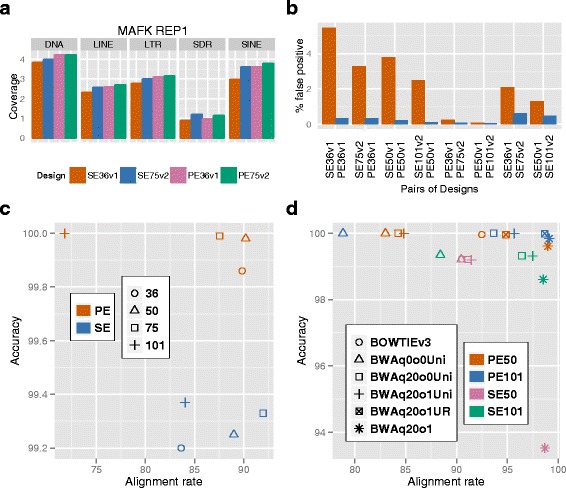
Alignment rate and accuracy of actual and simulated data. **a** Coverage of repeat elements in a MAFK dataset. Total lengths of the reads that overlapped repeat elements are normalized by the total lengths of the repeat elements. **b** False positive rates of a CTCF dataset based on the relaxed alignment set (BWAq20o1n8 on PE101). **c** and **d** Alignment rate vs. accuracy of simulation data for Bowtie and BWA, respectively. x and y axis are percentages of aligned reads and correctly aligned reads. Medians across five replicates are reported for each comparison

Repetitive elements are DNA sequences that occur in multiple copies throughout the genome. These copies can be either adjacent to each other or interspersed. Repetitive genomic elements are important for many biological processes including regulation of gene expression [32, 33]. We evaluated the coverage of different designs over the repetitive genomic elements, and found that both PE designs and long reads had higher repetitive element coverage in most cases. We specifically focused on satellite DNA, long interspersed nuclear elements (LINE), short interspersed nuclear elements (SINE), long terminal repeat (LTR) elements, and segmental duplication regions (SDR) in our evaluation. Figure 1a and Additional file 1: Figure S3 report the coverage (number of mapped bases) in these repetitive regions. For NONO and MAFK, we found that (1) PE designs had up to 46 % higher coverage over the repeats than SE with the same read length; (2) doubling the read-length (from 36 to 75 bps or from 50 to 101 bps) improved coverage for up to 35 % depending on the TF, read-length, and the type of repeats; and (3) the effect of long reads was larger for SE than PE. As an example, for one replicate of MAFK, the coverage over LINE elements of PE36v1 and SE75v2 were 11 % and 10 % more than that of SE36v1, respectively, and the coverage of PE75v2 was 3.9 % more than that of PE36v1 (Fig. 1a). For CTCF, PE designs and the long reads had much less or even no advantage, e.g., the coverage over LINE elements for SE75v2 was only 4.3 % more than that of SE36v1, and that of PE36v1 was even 2.3 % less than SE36v1 (Additional file 1: Figure S4a). We observed a trade-off between longer SE reads and short PE reads when comparing PE36v1 and SE75v2 at fixed number of sequenced bases. PE designs had higher (0–27 %) coverage over most repetitive elements for MAFK and NONO but not for CTCF (from 8.5 % lower to 2.6 % higher). On the other hand, SE designs had higher (up to 27 %) coverage over SDRs.

Thus, read length appeared to be a more critical parameter than the PE or SE aspect for improving coverage in SDR. Our results agreed with and complemented similar comparisons in the literature (e.g., Figure 2a in Chen et al. 2012 [11]). One reason for higher coverage of PE designs in repetitive regions is the increased mappability [34] of their reads. From this point of view, our results were consistent with the above numerical study, and also revealed that the tradeoff between read length and PE design in real data depends on the types of repetitive elements.

#### Accuracy of Bowtie alignments

False positives in alignment may cause false positives in detecting enrichment regions. We evaluated the alignment accuracy using both experimental data and fully simulated data, and found that both PE designs and longer reads improved alignment accuracy. We devised a strategy for evaluating alignment accuracy with the experimental data, even though the true origins of the reads were unknown. We used a relaxed alignment set, BWAq20o1n8 with the PE101 data as the “gold" standard, and labeled those reads that could not be aligned by this relaxed rule as false positives. Then we compared the false positive rates of the sequencing designs in pairs (e.g., SE36v1 vs PE36v1) and assessed the false positive rates in their alignment set differences (Fig. 1b). For example, the false positive rate of the reads that were aligned under SE36v1 but not PE36v1 was 5.47 %, and that of the reads aligned under PE36v1 but not SE36v1 was only 0.33 %. Our results in Fig. 1b showed that PE designs and longer reads led to lower false positive rates, and the advantage of longer reads was smaller for PE than for SE. Our simulation results, where the true origins of the simulated reads were used for measuring the accuracy, were consistent with the real data analysis (Fig. 1c and Additional file 1: Tables S15–S16). Figure 1c also highlighted a trade-off between the alignment rate and accuracy. Designs with 101 bps reads might have low alignment rates if the quality at the ends of the long reads are low, and the alignment rate of SE36 reads might be also low due to the lack of uniqueness. Other SE designs had high alignment rates, but low accuracy, and PE36, PE50, and PE75 had both high alignment rates and accuracy.

#### Coverage and accuracy of BWA alignments

BWA is also one of the most popular aligners for ChIP-seq data. Compared to Bowtie -v mode, it allows more flexible error control, and trimming of the poor terminal bases (option -q). It also reports one selected alignment location for reads aligned to multiple locations (multi-reads). While the majority of our analysis is based on Bowtie -v mode, we investigated a comprehensive set of alignment strategies based on BWA and compared them to the Bowtie -v mode in this section. This investigation also led to many practical suggestions on trimming (option -q) and filtering multi-reads in BWA alignment. In Table 2, where trimming of poor terminal bases was applied (option -q 20), we observed that the alignment rates under all designs were similar when all the multi-reads were kept. In contrast, when only the uniquely aligning reads were retained, both PE designs and long reads led to higher alignment rates. In our simulation study where we conducted a more comprehensive comparison, we observed a similar pattern (Fig. 1d and Additional file 1: Tables S15–S16). Additionally, our comparisons of Bowtie and BWA alignments indicated that they had almost the same coverage and accuracy when their alignment rules were comparable (e.g., for SE50, BOWTIEv3 and BWAq0o0n04Uni are essentially identical rules). In summary, both our data analysis and simulation experiments led to the following observations. Discarding multi-mapping reads in BWA output improved alignment accuracy, especially for SE designs. When such reads are retained, their inaccurate alignments were enriched in many regions with a potential impact on the downstream analysis (Additional file 1). When filtering multi-mapping reads from BWA output of PE data, only those pairs with multiple alignment locations for both ends needed to be removed, and those with one uniquely aligned end could be kept without loss in accuracy. Properly trimming by the -q argument in BWA increased coverage without loss in accuracy, especially for long SE reads. For example, trimming increased the alignment rate of SE101 from 88.41 % (BWAq0o0Uni) to 96.42 % (BWAq20o0Uni), while the accuracy remained almost the same (slight decrease from 99.35 % to 99.32 %) in the simulated data.

### The effect of read parameters on peak calling

#### Number of peaks by SPP+IDR

We next evaluated the impact of designs on the number of detected protein-DNA interactions, i.e., peaks. We adopted the robust ChIP-seq uniform processing pipeline [18] developed and extensively used by the ENCODE consortium. This pipeline first generates *relaxed peaks*, a list of approximately 3×10^5^ regions using peak caller SPP [2]. Then, the *optimal set of peaks* is selected as the subset of the relaxed peaks with better peak score than the threshold needed for controlling the irreproducible discovery rate (IDR) [25] at a given level.

**Fig. 2:**
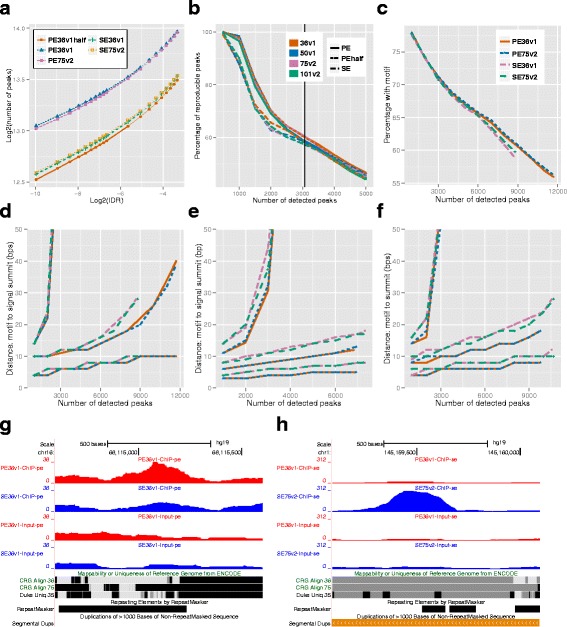
Effect of designs on peak calling. **a** Number of MAFK peaks by the SPP+IDR pipeline. **b** Reproducibility of the top SPP peaks of the simulated data. The black solid vertical line is the number of true peaks. **c** Percentages of the top SPP-identified MAFK peaks with the motif. **d**–**f** The 0.3, 0.5, and 0.7 quantiles of the distance between the motifs and the peak summits for the top ranked SPP peaks, MOSAiCS peaks and MACS2 for MAFK, respectively. Their legends are the same as in (**c**). **g**–**h** UCSC Genome browser view over two PE and SE specific BHLHE40 peaks with large coverage change. chr16:68155100 **g** was identified as a peak only with PE36v1 but not SE36v1, and chr1:145159593 **h** was identified only with SE75v2 but not PE36v1. The coverage tracks in each peak are normalized to the same scale. Lighter color on the mappability tracks indicates low mappability

We found that PE designs led to larger numbers of optimal peaks than SE designs regardless of the IDR level (Fig. 2a and Additional file 1: Figure S5). This was largely driven by the number of reads because SPP treats the two ends of PE reads as two SE reads, instead of processing the pairs as pairs. When the numbers of reads were controlled (e.g., PE36v1half versus SE36v1), the two designs resulted in comparable numbers of optimal peaks. Furthermore, read length had no noticeable impact on the numbers of peaks.

#### Reproducibility of peaks

Discovery from high throughput assays are often subject to variability across biological replicates, and, as a result, reproducibility of the discovered signals is a recurrent concern. The ChIP-seq uniform processing pipeline of ENCODE uses IDR to capture the reproducibility of the peaks between two replicates. In our simulations, we compared the designs using the reproducibility of the peaks across five replicates, and found that PE designs had better reproducibility. We measured the reproducibility by the minimax summit distances of the highest ranked peaks, which can be viewed as the maximal distance between the estimates of the same target in the five replicates. We labeled a peak as reproducible if its minimax summit distance was less than 200 bps, and higher proportion of reproducible peaks among the top peaks indicated better reproducibility. Overall, PE peaks were more reproducible than the SE peaks (Fig. 2b), which further elucidated why PE designs led to more peaks for fixed IDR levels.

#### Peaks in repetitive elements

In the previous section, we compared the designs in terms of their alignment coverage of repetitive elements. In this section, we revisited the repetitive regions, and investigated their peak coverage (percentages of peaks) under different designs. We overlapped the top peaks of each design with satellite DNA, LINE, SINE, LTR elements, and SDRs (Additional file 1: Figure S6). We compared these observations with those at the alignment level (Fig. 1a and Additional file 1: Figure S4), and found that the two did not necessarily exhibit the same pattern. For example, PE designs yielded higher coverage of MAFK reads in SINE elements (Additional file 1: Figures S4e,f) but smaller portion of MAFK peaks within the same type of repeats (Additional file 1: Figure S5o). In contrast, long reads led to both higher CTCF coverage and higher portion of CTCF peaks in SDR (Additional file 1: Figures S4a,b and Additional file 1: Figure S6j). We remark that, since PE designs identified more optimal peaks, they also yielded more peaks overlapping with repetitive elements than their SE counterparts, even if the overall proportion was slightly smaller.

#### Evaluation of the peak sets by motif occurrence and motif-to-summit resolution

The peaks of a sequence-specific DNA binding transcription factor typically harbor DNA motifs that the TF binds to. A commonly used metric for the quality of the peak lists is the proportion of the reported peaks with at least one motif. We found that a slightly higher proportion of the SPP peaks from PE designs harbor a motif than the SE peaks for MAFK, but not for CTCF or BHLHE40 (Fig. 2c and Additional file 1: Figure S7).

Another commonly used criteria for evaluating peak quality is their resolution, the distance between the summit of the peak and the nearest motif. A shorter distance between the motif and the summit indicates better resolution in peak calling. In our motif analysis, PE designs yielded slightly better resolution than SE for peaks of similar ranks for CTCF and MAFK (Fig. 2d and Additional file 1: Figure S8). The parameters that impact the peak-summit resolution include fragment size and numbers of correctly aligned reads. SPP estimates the fragment size with the same procedure for both SE and PE designs and utilizes a single estimated fragment size for all the reads. Although Chen et al. (2012) [11] showed that the SPP estimates of the fragment sizes are within 10–20 bps of the average value obtained from PE data, the use of a single fragment size discards the variation in the estimated fragment size, and SPP does not fully utilize the information of individual fragment lengths available in PE reads.

The majority of the currently available peak callers are developed for SE data, and only few of them are readily adapted for PE [5, 35, 36]. To evaluate the gain due to using read-specific fragment sizes which are readily available in PE data, we re-analyzed CTCF and MAFK datasets with MOSAiCS [5] and MACS2 [36]. MOSAiCS processes PE reads by acknowledging that every read pair represents a single fragment. We combined MOSAiCS with IDR to make the error control compatible with the uniform processing pipeline. For each peak, MOSAiCS has the capability of reporting two types of summits: a ChIP-seq signal-based summit and a sequence-based summit which incorporates sequence information into summit detection. We focused on the resolution analysis of the signal-based summit because these summits were more comparable to the summits reported by SPP. As expected, we observed a larger advantage of PE over SE in terms of motif-to-summit resolution (Fig. 2e and Additional file 1: Figure S9). MACS2 [36] also processes PE reads properly. We ran a similar analysis using MACS2 output with default parameters without IDR control, because we did not have a reliable pipeline that combines IDR and MACS2. Nevertheless, the MACS2 results were consistent with MOSAiCS results (Fig. 2f and Additional file 1: Figure S10). This analysis confirmed that fully leveraging the PE designs might improve peak calling in terms of resolution.

#### Analysis of peak set differences

The optimal peak sets reported under different designs were highly similar (overlap between any two >85 %). Such consistency indicated that all designs targeted the same overall signal pattern. In this section, we focused on the peak set differences, and investigated to what extent the differences could be attributed to the change in the ranking of the relaxed peaks and the alignment differences that led to significant changes in coverage. We first used SE36v1 as the baseline and investigated the changes in peak ranking and peak region coverage when switching to PE36v1 or doubling the read-length (SE75v2). Additional file 1: Tables S17–S19 summarize the results of these comparisons. For each pair of designs under investigation (e.g., PE36v1 vs. SE36v1), we overlapped their top *M* peaks, i.e., two peaks were declared as overlapping if their summit-to-summit distance ≤ 200 bps, treated the overlapping peaks as the estimates of the same target, and compared their ranks. For each TF, we chose nine M values ranging from 12 % to 120 % of the size of its optimal peak list. For example, among the top 80,000 CTCF peaks under PE36v1, 75,354 of them overlapped with the top 80,000 SE36v1 peaks, another 3,181 peaks overlapped with the the SE36v1 peaks ranked between 80,000 and 1.2 ×80,000, another 767 overlapped with SE36v1 peaks ranked between 1.2 ×80,000 and 3 ×10^5^, and the remaining 48 did not overlap with any of the SE36v1 relaxed peak. This comparison indicated that the ranks of the 75,354 of the top 80,000 PE36v1 peaks effectively remained unchanged under SE36v1 (still among the top 80,000 peaks under SE36v1), 3,181 of them underwent moderate rank change (still in the top 1.2 ×80,000), 767 of them suffered from large changes in rank, and at most 48 of them were specific to PE36v1, and could not be recovered under SE36v1. In summary, we observed that (1) more than 80 *%* of the ranks effectively remained unchanged in any comparison; (2) the majority of the rank changes were moderate; and (3) only a small portion (usually less than 2 %) of the peaks seemed specific to one design, with the exception of BHLHE40, where 10–15 % of the SE peaks were specific to the SE designs. We focused on the peaks in the last category (specific to one design), and further examined their ChIP and Input coverage. Surprisingly, we found that even for these peaks, the coverage around the summits were usually similar in different designs, and only a small percentage (≤50 % except for one case) exhibited a fold-change of at least 1.5 in any replicate of ChIP or Input samples (Additional file 1: Table S20). Thus, if more than 3×10^5^ peaks were included in the relaxed peak list, an even larger portion of peak set differences could be explained by rank change. We next investigated the motif occurrence and repetitive element coverage of the design-specific peaks with at least 1.5 fold-change in coverage between the designs (Additional file 1: Tables S21–S23). We observed that there were barely any motifs except in one case (SE75v2 specific peaks when comparing with PE36v1), and SDR were enriched for all the peaks that were specific to PE or long read designs. We visualized two such BHLHE40 peaks with motifs in the UCSC Genome Browser, and found that both were in low mappability regions (Fig. 2g,h and Additional file 1: Figure S11).

### The effect of read parameters on allele-specific binding detection

#### Allele-specific binding detection by AlleleSeq

Allele-specific binding (ASB) detection searches for differences in TF binding between the two alleles of the same individual at a given set of loci (SNPs). ASB studies provide insights in understanding genomic imprinting [37] and non-coding disease variants [38], and a well-controlled model for understanding the population effects of functional variations in TF binding [8] and other epigenomic processes [9]. Although there are currently a number of methods for ASB detection [8, 9], the impact of design on ASB detection has not been studied. In the following comparison, we utilized the current AlleleSeq [8] pipeline for SE designs and adapted it to the PE designs.

#### Numbers of ASB loci detected by different designs

**Fig. 3:**
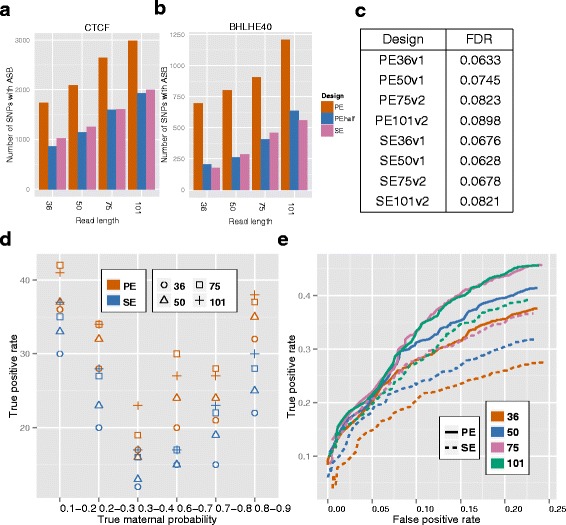
Effect of designs on ASB detection. **a**–**b** Numbers of SNPs that exhibited ASB in the CTCF and BHLHE40 datasets. **c** Empirical FDR of ASB detection in the simulated data. **d** Percentages of ASB exhibiting SNPs detected by AlleleSeq in the simulation study (*p*-value ≤0.05) under different designs. **e** ROC curve of ASB detection on simulated data. The reported values are averages over five replicates

We compared the numbers of ASB loci detected by different designs at AlleleSeq default FDR level of 0.1 and observed an increasing trend with the number of sequenced bases (Fig. 3a,b). At fixed number of sequenced fragments, i.e., PE versus SE comparisons, longer reads detected more ASB loci, and PE designs yielded 1.4 to 4 times more detected ASB loci than SE with the same read-length. When both the number of fragments and the number of sequenced bases were similar (e.g., PE36v1 vs SE75v2), PE designs yielded 7 to 11 % more detected ASB loci for CTCF, and 42–51 % more for BHLHE40. At fixed read-length, PEhalf designs, which have half of the fragments of SE designs, were overall comparable to the SE designs in the numbers of detected ASB loci.

#### Sensitivity and specificity of ASB detection

We next assessed the impact of sequencing designs on the sensitivity and specificity of ASB detection with a simulation study. We applied AlleleSeq on the simulated data and thresholded the *p*-values at 0.05 to compute the empirical FDR. We found that PE designs and longer reads had higher true positive rates regardless the range of the true maternal probability (Fig. 3d), at the expense of slightly higher FDR levels (Fig. 3c). We then evaluated the true and false positive trade-off with an ROC curve (Fig. 3e), and observed that (1) the differences in performances were, to a large extent, driven by the number of sequenced bases; hence, long reads performed better than short reads, and PE designs better than the SE designs with the same read-length; (2) the advantage of long reads over short reads was larger for SE than for PE, and the 101 bps reads did not perform much better than 75 bps reads; and (3) when the number of sequenced bases was controlled, PE50 performed better than SE101 and the performances of PE36 and SE75 were similar.

#### Consistency among designs in ASB detection

**Fig. 4:**
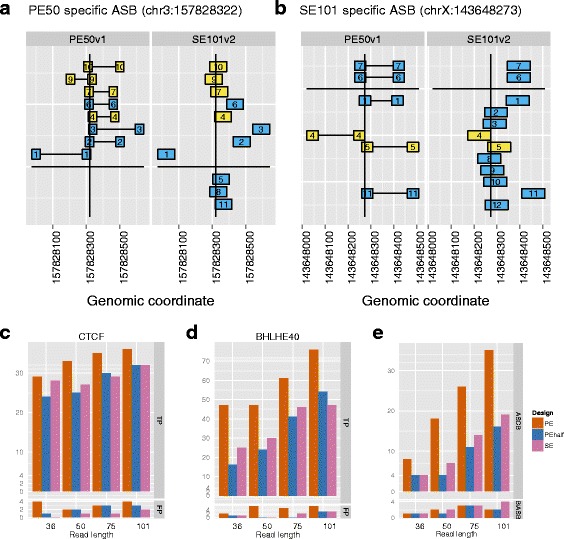
Effect of designs on ASB detection (cont). **a**–**b** Aligned reads at two SNPs with design-specific ASB for CTCF. chr3:157828322 exhibits ASB only under PE50v1 but not SE101v2, and vice versa for chrX:143648273. The vertical lines indicate the SNP location on the reference genome. Yellow rectangles are the reads that are properly mapped and cover the SNP under both designs. Blue rectangles are the reads that are mapped only under one design, or are mapped under both designs, but only cover the SNP under one design, but not the other. The two ends of the same fragments are connected via horizontal lines for the PE design. In each figure, the PE and SE reads labeled with the same number are from the same fragment. If one number only appears under one design, this read must have been filtered out or could not have been mapped under the other design. The reads assigned to the maternal allele are above the long horizontal line, and those assigned to the paternal allele are below the horizontal line. AlleleSeq only counts the reads overlapping with the SNP (the vertical line) when testing the allelic imbalance. **c**–**d** Numbers of SNPs with ASB and ASOC in favor of the same allele (TP) and different alleles (FP) for CTCF and BHLHE40 datasets, respectively. **e** Numbers of ASCB and BiASB of CTCF and BHLHE40 datasets

When we overlapped the set of ASB loci identified by different designs, we observed low overlapping rates for many comparisons (Additional file 1: Figure S12), e.g., AlleleSeq identified 2086 ASB loci for CTCF under PE50v1, and 1994 ASB under SE101v2, and only 1356 of them were identical, roughly a 2/3 overlapping rate. Figure 4a,b provide illustrative examples of PE50v1 specific and SE101v2 specific ASB events, respectively. In these two figures, some reads were only mapped and covered the SNP under one design (blue rectangles). Others were mapped in both designs and covered the SNP (yellow rectangles). Although both designs had low coverage in this example, low coverage was not generally correlated with the differences in ASB detection. In fact, the overall read counts at the ASB loci that were identified only by PE50v1 (or SE101v2) were comparable to all ASB detected under each design (Additional file 1: Table S24).

#### Motif comparison of the two alleles at the ASB loci

We next evaluated the accuracy of ASB detection in the CTCF dataset using motif information, and found that PE and long reads led to higher detection accuracy. Specifically, we compared the *p*-values of the FIMO [28] reported matches to the CTCF motif in the winning, and losing alleles. If the *p*-value of the motif match in the winning allele was smaller than that of the losing allele, this ASB loci was deemed more likely to be a true positive because the winning allele supported binding with a better motif match. However, we did not expect the ASB loci without this property to be false positives because ASB might be manifesting itself through other factors such as open chromatin structure and binding of co-factors. We considered the intersection and set differences of the ASB loci for each pair of designs under investigation (e.g., PE50v1 and SE101v2) and compared FIMO *p*-values of the motif matches in both alleles (Additional file 1: Figure S13). We further reported the number of ASB loci with a motif in both alleles, only in the winning, only in the losing allele, and in neither of the alleles in Additional file 1: Table S25 using the default threshold for the FIMO *p*-values (<0.0001). In summary, we concluded that SE75v2 design was better than PE36v1 in ASB detection accuracy. This is largely because, for the ASB loci only identified under SE75v2 but not PE36v1, the overall improvement of the motif score in the winning allele over the losing allele was larger than those that were only identified under PE36v1 (Additional file 1: Figure S12a). Furthermore, 11 of the ASB loci that were only identified under the PE36v1 design but not SE75v2 design had a motif only in the winning allele, and 8 of them had one only in the losing allele. In contrast, the set of ASB loci specific to SE75v2 had 15 loci with motif only in the winning allele and one locus with motif only in the losing allele (Additional file 1: Table S25). These analysis further indicated that (1) for the same read-length, PE designs performed better than SE; (2) long reads performed better than short reads for both PE and SE, with a possible exception of PE101v2 vs. PE50v1; and (3) when the number of sequenced bases were controlled, PE36v1 under-performed compared to SE75v2, but PE50v1 was better than SE101v2. Overall, the best design from this perspective was among PE50v1, PE75v2, and PE101v2; and in each pairwise comparison, the ASB loci identified under both designs were better than the others that are specific to one design in terms of accuracy.

#### Consistency between DNase-seq and ChIP-seq in allele-specific behavior

DNase-seq experiments elucidate broader regions of open chromatin which often exhibit transcription factor occupancy [39–41]. Therefore, it is natural to expect interactions between allele-specific open chromatin (ASOC) structure and ASB. Towards this end, we examined the consistency between the detected allele-specific behavior from DNase-seq [42] and ChIP-seq data from the same cell line. We identified a conservative list of loci with allele-specific behavior in DNase-seq as high confidence allele-specific open chromatin (ASOC) regions. We expected these loci to be ASB loci in favor of the same allele if they also overlapped ChIP-seq peak regions. There were 94 and 153 loci with ASOC in CTCF and BHLHE40 peaks, respectively. Figure 4c,d evaluate the sensitivity of ASB detection by the number of loci with both ASOC and ASB in favor of the same allele, and the detection errors by the number of loci with ASOC and ASB in favor of different alleles. For the same read-length, PE designs had higher sensitivity than SE designs, at the expense of slightly elevated error levels. The sensitivity gain for CTCF ranged in 3–22 % for CTCF, and in 32–88 % for BHLHE40. In both cases, the gain was driven by the number of sequenced bases. When the number of bases were controlled (e.g., PE36v1 vs. SE75v2), PE and SE designs performed similarly.

#### Allele-specific co-binding of CTCF and BHLHE40

We defined allele-specific co-binding (ASCB) events as the peak regions where two or more TF bound in favor of the same allele. In contrast, we defined the peaks where binding by those factors favored different alleles as bi-allele-specific binding (BiASB). Partially due to the interaction between binding and open chromatin structure, we expected ASCB to be more common than BiASB. We then investigated ASB behaviors of CTCF and BHLHE40 in their co-binding regions. Similar to the last section, we adopted a more conservative criteria for ASB detection. We observed that the designs with similar amounts of sequenced bases yielded similar numbers of ASCB and BiASB, and the numbers of BiASB were much smaller than the numbers of ASCB as expected (Fig. 4e).

## Conclusions

The impact of sequencing design on the downstream analysis is profound and complicated. It relies heavily on research goals, data quality, and the computational tools chosen for the analysis. Our results suggest that both PE designs and long reads improve the alignment accuracy of Bowtie and coverage in repetitive regions. The trade-off between PE designs and long reads depend on the expected data quality. For peak calling, PE designs yield more peaks with comparable quality (in terms of motif occurrence and resolution) to their SE counterparts, and the quality of PE peaks can be further improved if the peak caller is able to properly process PE information. On the other hand, read-length does not have much effect in peak calling. For ASB detection, both PE designs and long reads lead to more detected ASB with higher accuracy.

Our computational and data-driven experiments support PE designs for their higher alignment accuracy and higher coverage in repetitive elements especially for TFs where the antibody or other experimental conditions are far from ideal. We did not observe a clear advantage of read length of 101 bps. PE36 worked as good as other PE designs for peak calling and PE50 and PE75 worked best for ASB. One reviewer suggested that the observed design differences may depend on the particular error profiles of the data used. While this could be true, the typical error profiles from Illumina platforms have been well characterized and lower quality near the 3^′^ end of reads is commonly seen [31]. Thus the insights we learned in this study could be generalized with reasonable caution. In addition, variation in read error profiles is not the only factor that may affect the generality of the studies on sequencing design. Numerous technical and human factors are potential contributors.

While it is up to the individual investigator to decide whether the potential improvement in biological findings is worth the costs of the more expensive designs, this study provides a computational perspective and highlights the importance of using appropriate computational tools to maximize the power of the chosen design. For BWA alignments, we found that filtering improves the alignment accuracy, especially for SE and short reads, and trimming by option -q improves the coverage in aligning the long reads. However, despite the evidence presented in this paper and in the literature [31], we acknowledge that trimming could be a contentious topic due to the potential bias it introduces in read density. When identifying peaks from PE designs, we suggest using a peak caller that fully leverages the paired-end information of the data. Without using appropriate computational tools in the analysis protocol, the extra cost of paired-end ChIP-Seq experiments may not be justified.

We systematically investigated the design effects using *in silico* data generated from ChIP-seq experimental data, instead of comparing publicly available ChIP-seq experiments using the same antibody and the cell line, but different designs. This is because our approach avoids the unnecessary variations in ChIP-seq experiments performed by different investigators using different facilities at different times. For example, ENCODE PE data are generally two to three years newer than their SE counterparts. In addition, generating *in silico* data via subsampling and trimming methods is common practice in the literature [11, 12]. Hence it is reasonable to expect similar conclusions being drawn from the comparisons of the ChIP-seq experiments performed in exactly the same way except the read designs. We only considered Bowtie and BWA as aligners in our current study because they are currently the most commonly used read mappers in ChIP-seq analysis. We mainly used SPP for peak calling, because it works well with IDR, which allowed us to assess reproducibility. The majority of the popular peak detection tools (including SPP) do not process PE information appropriately. Thus we only included MOSAiCS and MACS2, two representative peak callers with such capability, instead of comparing all popular peak-calling tools. We focused on ChIP-seq experiments for transcription factors, and designed and adopted systematic evaluation criteria. Interesting extensions include cross-species comparisons of the design effects, and investigating the sequencing design effects on ChIP-seq experiments for histone modifications, and other experiments such as Bisulfite sequencing [43], and 5-hmC sequencing [44].

The impact of sequencing depth on the detection of enrichment regions and many other factors that affect alignment and peak calling in the context of ChIP-seq have been investigated by others [11, 12]. Our work extends the current literature in multiple important perspectives by focusing on the sequencing design parameters, considering multiple alignment strategies and evaluating the combinations of design and alignment strategies. Most importantly, our study represents the first systematic evaluation of the impact of ChIP-seq designs on ASB detection and highlights the power of PE designs for ASB detection.

## Additional file

Additional file 1 **Supplementary materials.** The file that contains all supplementary notes, figures and tables. (PDF 1330 kb)
