# Supplementary material for: Systematic evaluation of the impact of ChIP-seq read designs on genome coverage, peak identification, and allele-specific binding detection
Source: BMC Bioinformatics. 2016 Feb 24;17:96. doi: 10.1186/s12859-016-0957-1 (PMC4765064; doi:10.1186/s12859-016-0957-1)
Supplement: Supplementary file 1 — Supplementary materials. The file that contains all supplementary notes, figures and tables. (PDF 1330 kb) [file 12859_2016_957_MOESM1_ESM.pdf]

Supplementary Materials of Systematic evaluation of the impact of ChIP-seq read designs on genome coverage, peak identification, and allele-specific binding detection

Qi Zhang [a,1], Xin Zeng[b], Sam Younkin[c], Trupti Kawli[d], Michael P. Snyder [d,e], Sündüz Keleş [b,c,2]

a Department of Statistics, University of Nebraska Lincoln, Lincoln, NE, USA

b Department of Statistics, University of Wisconsin, Madison, WI, USA

c Department of Biostatistics and Medical Informatics, University of Wisconsin, Madison, WI, USA

d Department of Genetics, Stanford University School of Medicine, Palo Alto, CA, USA

e Stanford Center for Genomics and Personalized Medicine, Palo Alto, CA, USA

Supplementary Notes

Regions enriched of inaccurate alignments

Our simulation study revealed that falsely aligned reads can be locally clustered, indicating enrichment in the ChIP sample. Such regions may lead to false positives in peak calling. We quantified the prevalence of these regions as follows. We divided chr19 into non-overlapping intervals of size 200 bps and counted the number of such intervals with at least 20 reads, 50% or more of which were aligned incorrectly. Supplementary Tables S26 summarized the medians of the number of such intervals over five replicates for all the alignment strategies we considered.

[1] Qi Zhang performed the research when he was a postdoctoral research associate under the supervision of Prof. Sündüz Keleş in Department of Biostatistics and Medical Informatics at University of Wisconsin Madison.

[2] Corresponding author. E-mail: keles@stat.wisc.edu

Supplementary Figures

Materials and methods

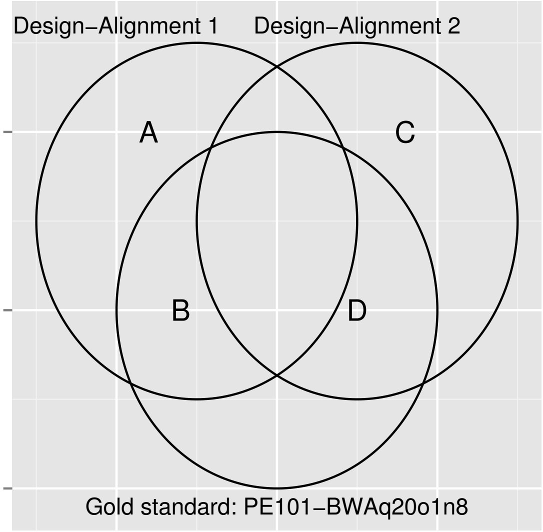

Supplementary Figure 1. Venn diagram illustrating the rationale of the alignment comparison in real data analysis using the "gold" standard alignment set. We only focus on the alignment difference of Design-Alignment 1 and Design-Alignment 2 (e.g., PE36v1 and SE75v2). The false positive rate of Design-Alignment 1 is $A/(A+B)$, and that for Design-Alignment 2 is $C/(C+D)$.

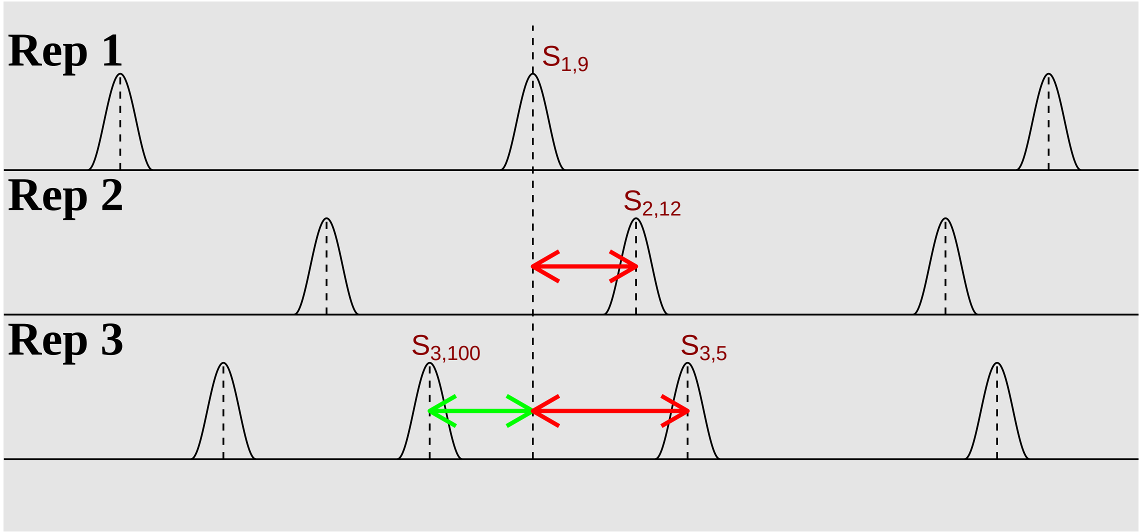

Supplementary Figure 2. Measuring the reproducibility of top ranked peaks across more than two replicates by minimax summit distance. This figure provides an illustration of how to measure the reproducibility of top ranked peaks across more than two replicates using minimax summit distance. Suppose there are three replicates of ChIP-seq data, and we obtain a ranked peak list from each replicate. Next, consider measuring the reproducibility of the top rank peaks, for example, the reproducibility of the top ten peaks across the three replicates. Since there will be variations among the peak sets of different replicates, we do not expect the top ten peaks to be the same in all replicates. However, if the top ten peaks in one replicate can be reproduced by, for example, the top fifteen (1.5×10) peaks in another replicate, it could be still considered as reproducible. In the figure, let us first focus on one peak from one replicate, e.g., the rank 9 peak from Rep 1, and let $S_{1,9}$ be its summit. In Rep 2, $S_{2,12}$ (the summit of the rank 12 peak from Rep 2) is the closest summit to $S_{1,9}$ in Rep 2. If the distance between $S_{1,9}$ and $S_{2,12}$ is short, it indicates that $S_{1,9}$ is reproduced in Rep 2 by $S_{2,12}$. Similarly, $S_{3,100}$ is the closest summit to $S_{1,9}$ from Rep 3. However, it is in the rank 100 peak, which is too low. Hence we move on to $S_{3,5}$, the summit closest to $S_{1,9}$ in the top fifteen peaks from Rep 3. If both of $S_{2,12}$ and $S_{3,5}$ are close to $S_{1,9}$, it indicates that $S_{1,9}$ is reproducible in all replicates. This is why we use their maximum (the maximum of the distances annotated by red arrows) to measure the reproducibility of $S_{1,9}$. We repeat this calculation for the rank 9 peaks from all three replicates ($S_{1,9}$,$S_{2,9}$ and $S_{3,9}$), and report their maximum as $L_{10}(9)$. If $L_{10}(9)$ is small, i.e., $\leq 200bps$, we label the rank 9 peaks as reproducible across all replicates. Finally, we measure the reproducibility of the top ten peaks across all replicates by the proportion of reproducible peaks.

Effect of design on read alignment

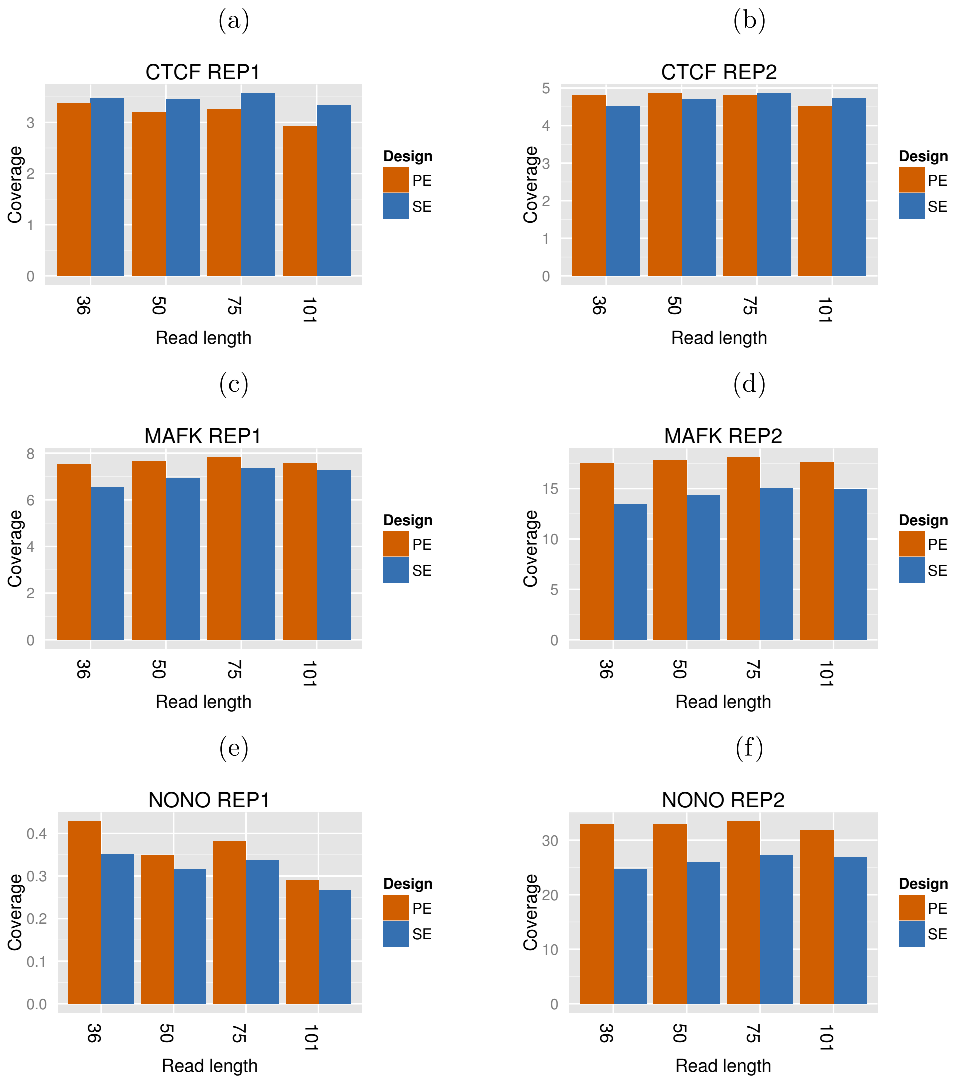

Supplementary Figure 3. Multi-coverage of various designs. Multi-coverage represents the percentage of the genome covered by at least five reads.

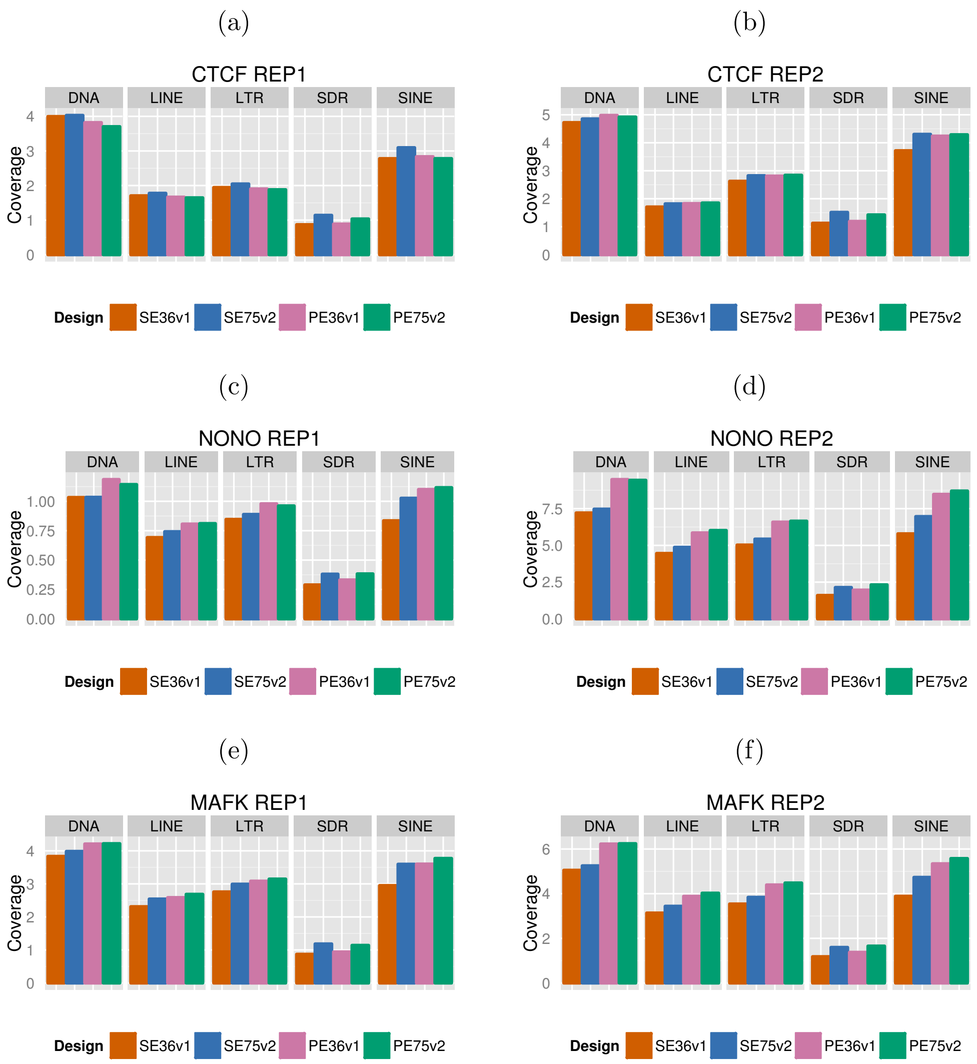

Supplementary Figure 4. Normalized coverage (total aligned bases in repetitive region/size of repetitive region) over different classes of repetitive elements for GM12878 CTCF and NONO and MCF7 MAFK. DNA stands for satellite DNA elements. The panel (e) is a replicate of Figure 1b in the main text.

Effect of design on peak calling

(a) CTCF **(b)** BHLHE40 **(c)** MAFK

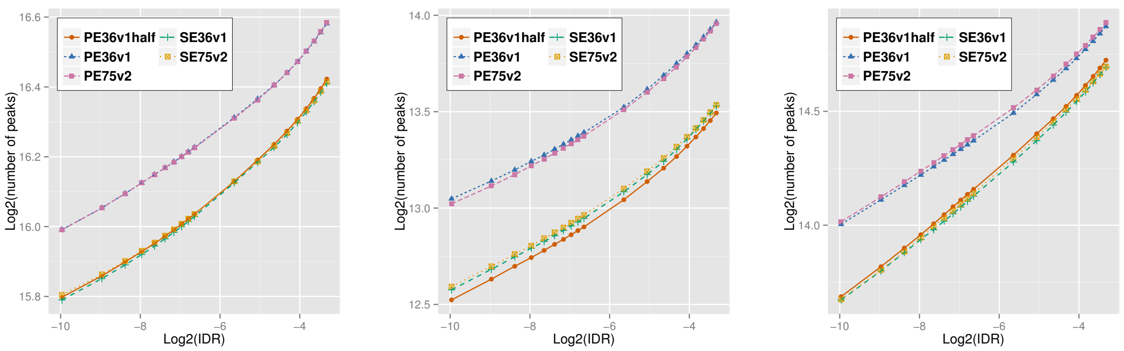

Supplementary Figure 5. Comparison of the numbers of peaks identified by the SPP+IDR pipeline under different designs. (c) is a replicate of Figure 2a in the main text

(a) CTCF

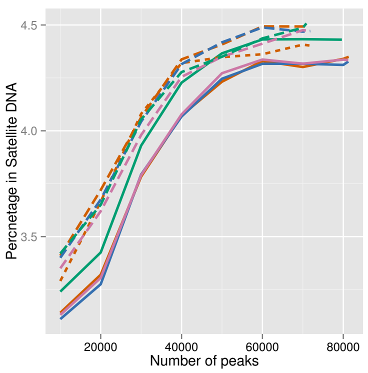

(b) BHLHE40

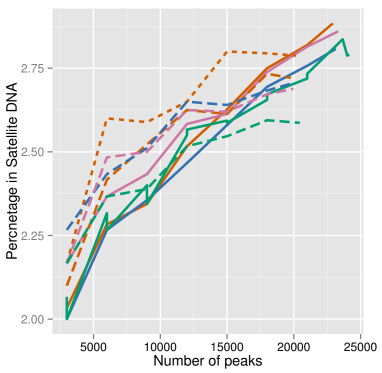

(c) MAFK

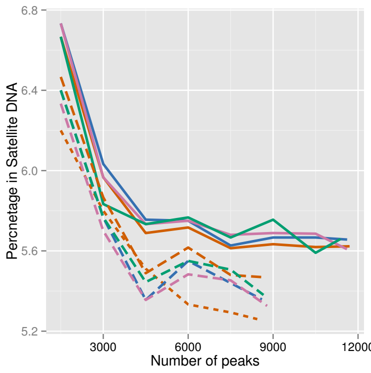

(d) CTCF

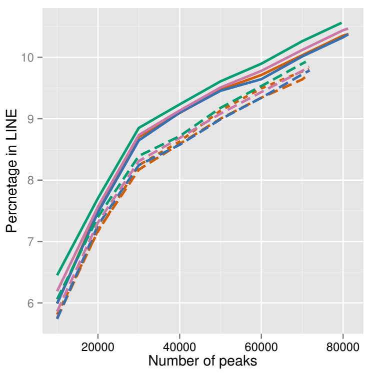

(e) BHLHE40

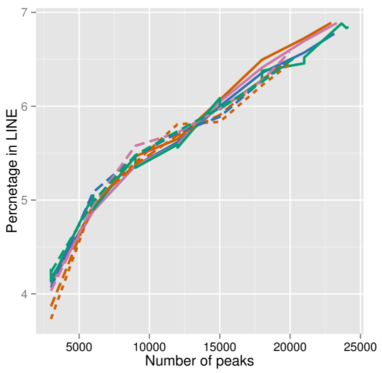

(f) MAFK

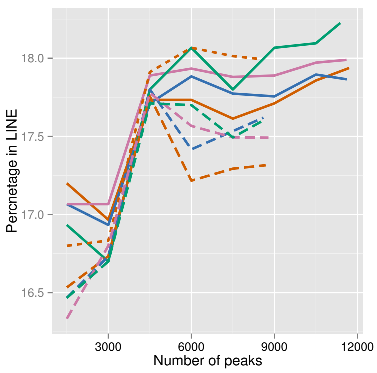

(g) CTCF

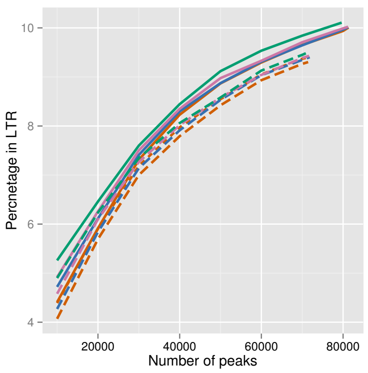

(h) BHLHE40

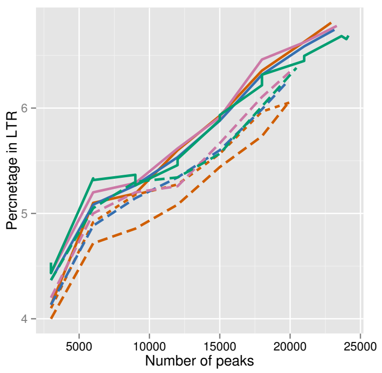

(i) MAFK

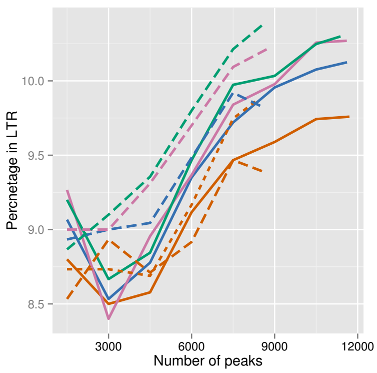

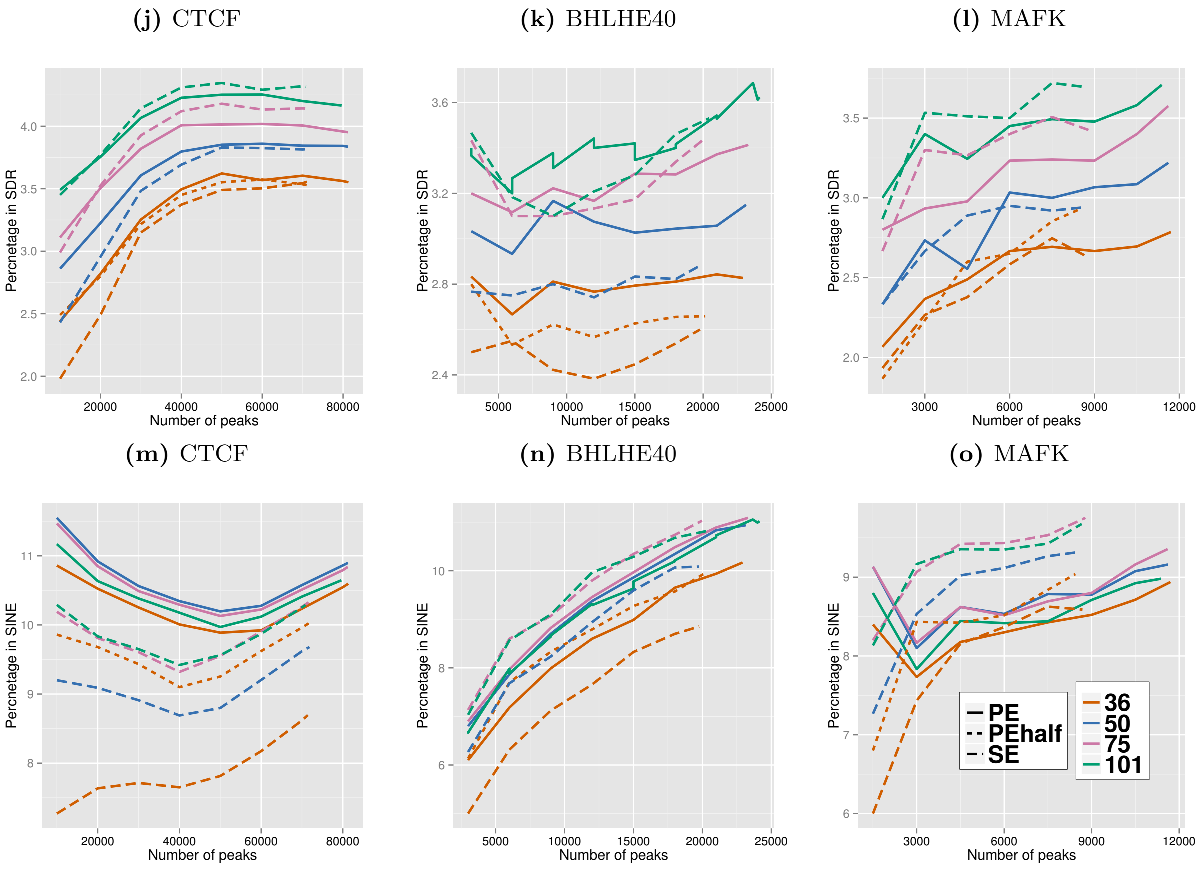

Supplementary Figure 6. Percentages of the top ranked SPP peaks that overlap with repetitive elements. The overlap analysis required at least 30% of the peak region to be located within the repetitive element.

(a) CTCF (b) BHLHE40 (c) MAFK

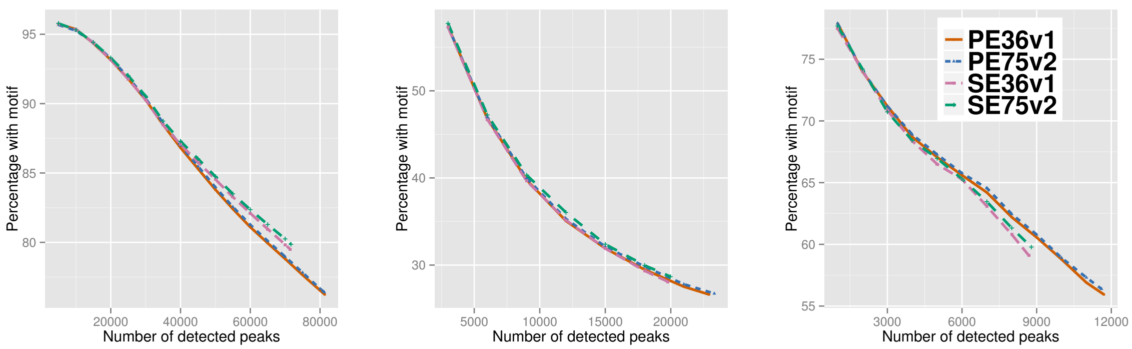

Supplementary Figure 7. Percentages of the top ranked SPP peaks with the motif. (c) is a replicate of Figure 2c.

(a) CTCF **(b)** MAFK

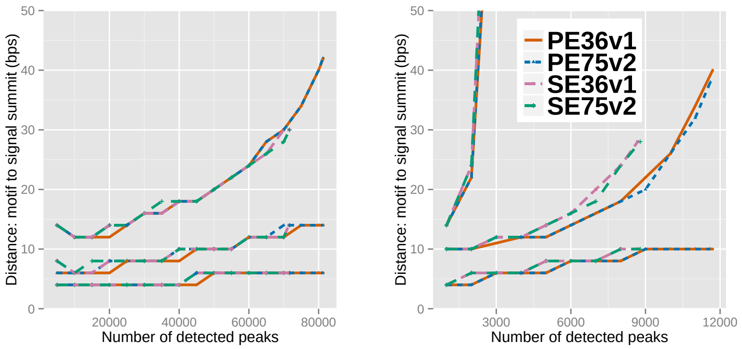

Supplementary Figure 8. The 0.3,0.5, and 0.7 quantiles (from bottom to top) of the distance between the motifs and the peak summits for the top ranked SPP peaks. For a peak with no motif, such distance is infinity, which is why some lines abruptly stop at certain points. (b) is a replicate of Figure 2d. The motif occurrence rates for BHLHE40 were very low ($< 50\%$) and hence are not displayed.

(a) CTCF **(b)** MAFK

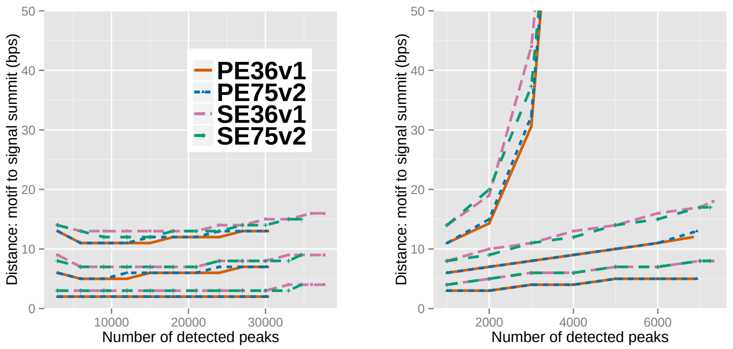

Supplementary Figure 9. The 0.3, 0.5, and 0.7 quantiles (from bottom to top) of the distance between the motifs and the signal summits for the top ranked MOSAiCS peaks. (b) is a replicate of Figure 2e. For a peak with no motif, such distance is infinity, which is why some lines stop abruptly at certain points.

(a) CTCF **(b)** MAFK

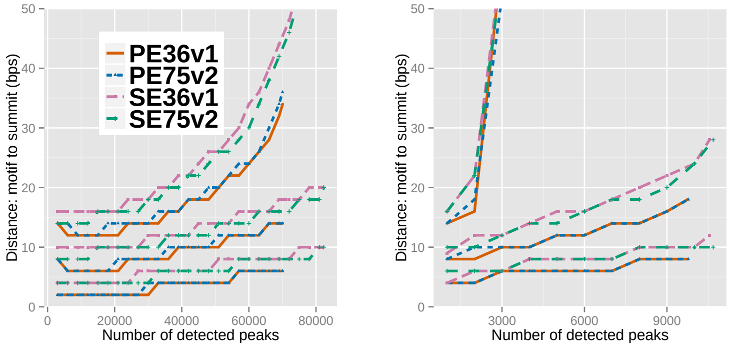

Supplementary Figure 10. The 0.3, 0.5, and 0.7 quantiles (from bottom to top) of the distance between the motifs and the signal summits for the top ranked MACS2 peaks. (b) is a replicate of Figure 2f. For a peak with no motif, such distance is infinity, which is why some lines stop abruptly at certain points.

(a) A PE36v1 peak that cannot be recovered under SE36v1. The summit is chr16:68115100

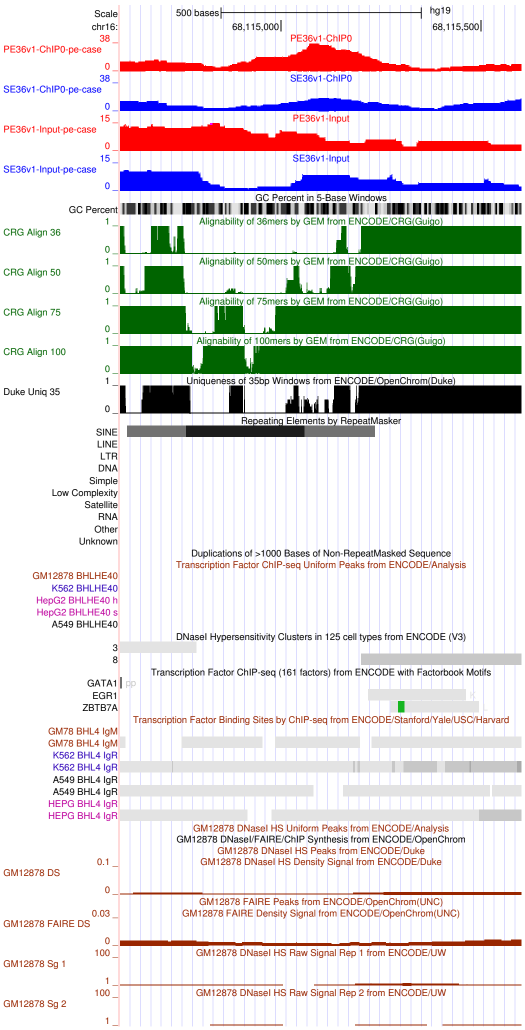

(b) An SE75v2 peak that cannot be recovered under PE36v1. The summit is chr1:145159593

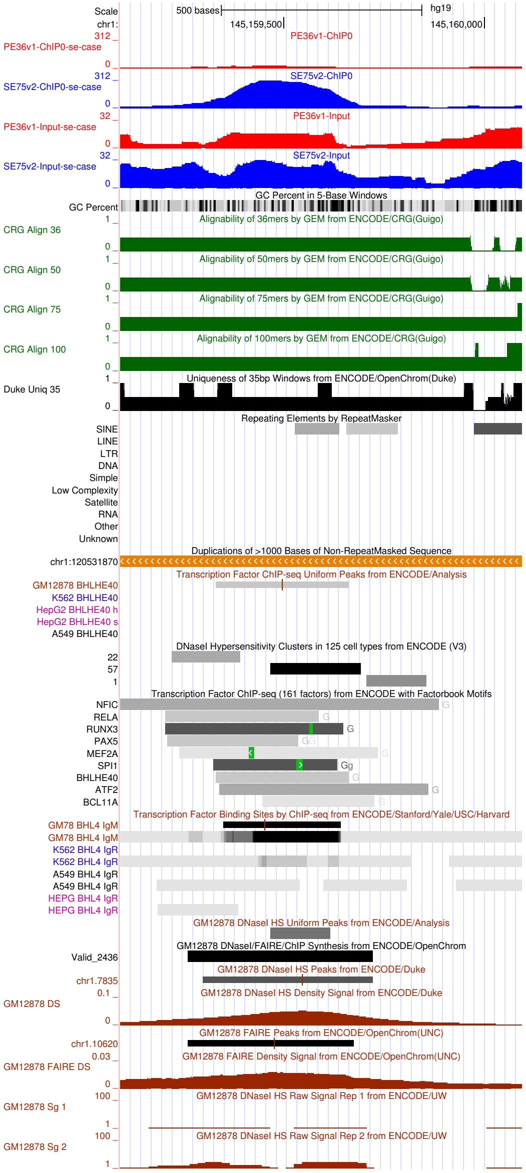

Supplementary Figure 11. The UCSC Genome Browser views of two BHLHE40 peaks that are specific to one design and cannot be recovered by the other. For each peak, the summit is at the center of the region, and the 1000 bps window centering the summit location is displayed. The coverages over the summit under the two designs have at least a 1.5 fold-change in ChIP and/or Input for both of the peaks, and both peaks harbor a BHLHE40 motif. Both peaks appear to be in repetitive regions with low mappability. The SE75v2 peak is also supported by the open chromatin structure and ChIP-seq peaks from ENCODE, which might be because these ENCODE peaks were identified from data with SE designs.

Effetc of design on ASB detection

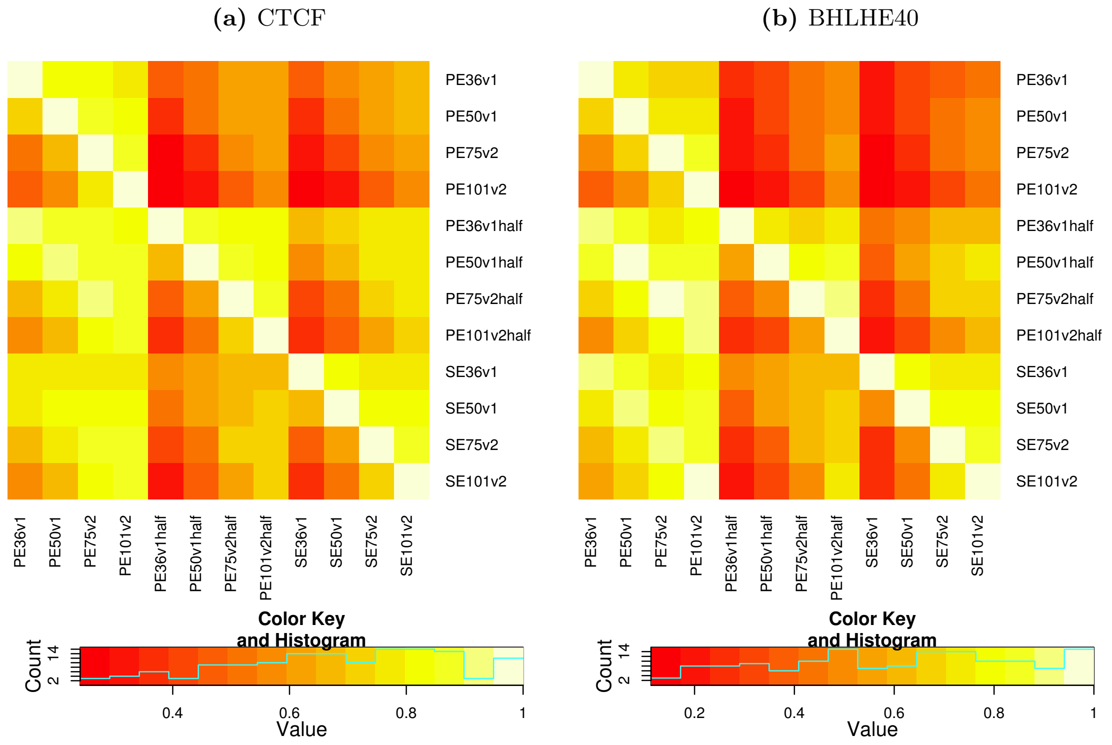

Supplementary Figure 12. Comparison of AlleleSeq results under different designs. Each entry displays the overlap proportion between the ASB events detected by different designs. Overlap is computed as the size of the intersection set divided by the size of the set in the row.

(a) PE36v1 vs SE75v2

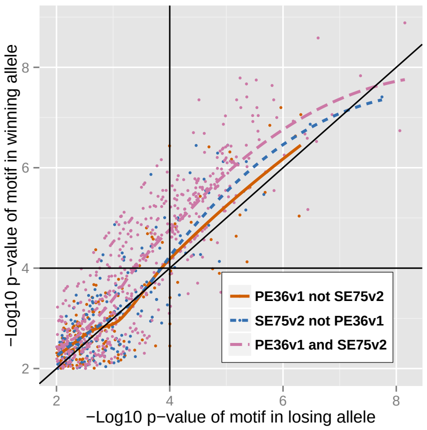

(b) PE50v1 vs SE101v2

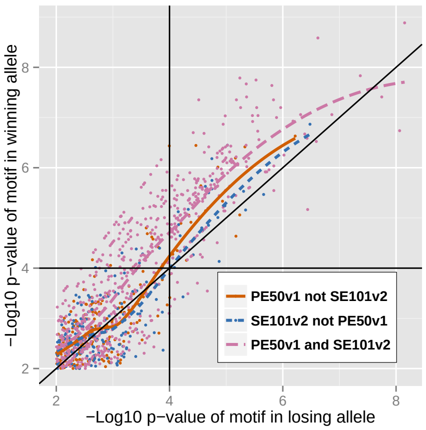

(c) PE36v1 vs SE36v1

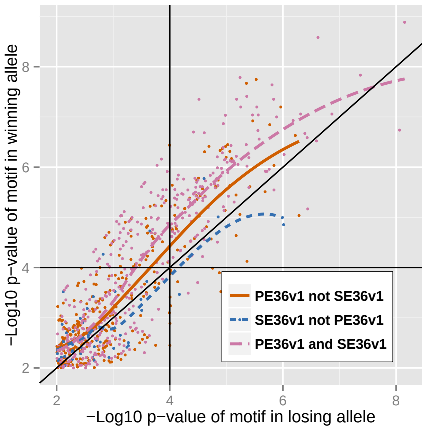

(d) PE50v1 vs SE50v1

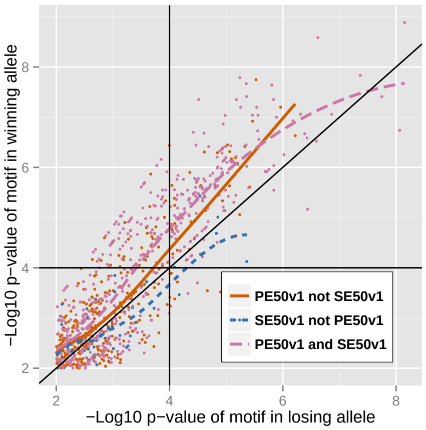

(e) PE75v2 vs SE75v2

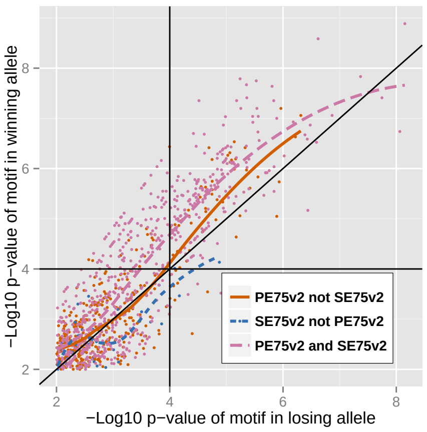

(f) PE101v2 vs SE101v2

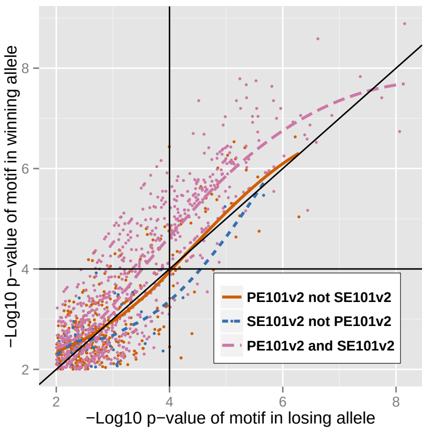

(g) SE75v2 vs SE36v1

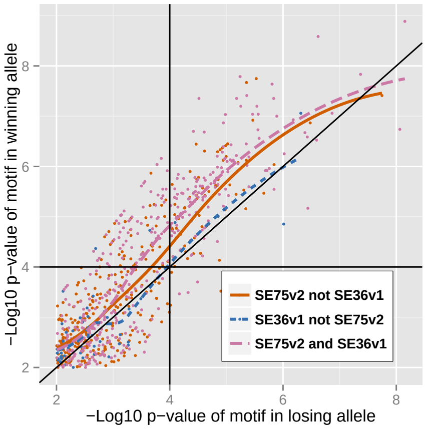

(h) SE101v2 vs SE50v1

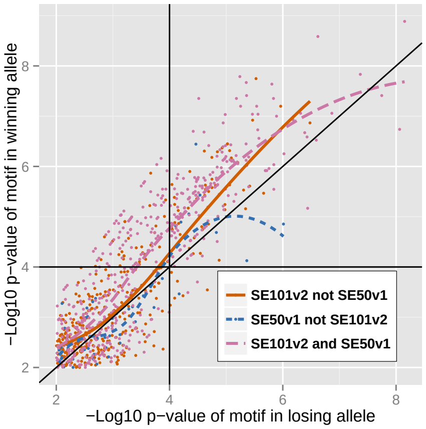

(i) PE75v2 vs PE36v1

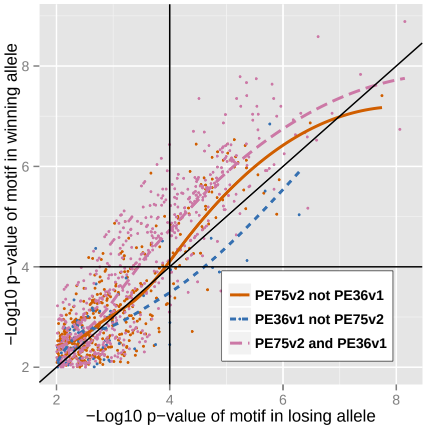

(j) PE101v2 vs PE50v1

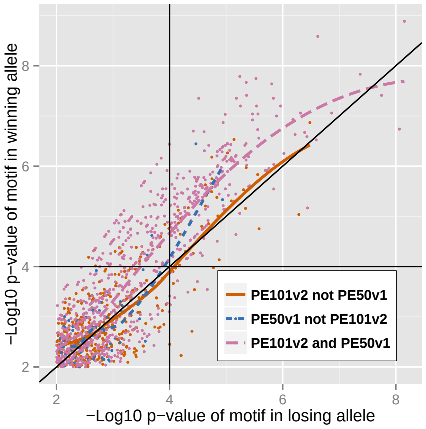

Supplementary Figure 13. Comparison CTCF motif matches in the winning and losing alleles at the detected ASB loci of the CTCF dataset. ASB loci for each pair of designs were divided into three groups as the common set and the set differences. For the common ASB loci, the winning alleles were the same under both designs. The minus log base 10 of the p-values from FIMO and the corresponding lowess curves are displayed for each group using SNPs with matches with p-value<0.01 on both alleles. The others are less relevant, because motif occurrences were determined with a default p-value cut-off of 0.0001. The points above the line $y = x$ represent ASB instances where the CTCF motif score of the winning allele is higher than that of the losing allele, which indicates that the ASB detection at this SNP is supported by motif evidence. The higher the points/lowess lines are, the stronger the evidence is. The vertical and horizontal lines are at the default FIMO p-value cutoff of 0.0001. The points in the upper left quadrant are those SNPs where only the winning allele has a motif (p-value<0.0001) and those in the lower right quadrant vice versa.

Supplementary Tables

Materials and Methods

Supplementary Table 1. Numbers of interrogated fragments in each sample.

Sample	Fragments
GM12878 CTCF REP1	24318340
GM12878 CTCF REP2	28270343
GM12878 BHLHE40 (DEC1) REP1	41514047
GM12878 BHLHE40 (DEC1) REP2	41708224
GM12878 NONO REP1	15021338
GM12878 NONO REP2	52740748
MCF7 MAFK REP1	27708627
MCF7 MAFK REP2	36685798
GM12878 Input	56163459
MCF7 Input	79438117

Numbers of interrogated fragments in each sample. These numbers correspond to numbers of reads for SE designs and numbers of read pairs for PE designs.

Supplementary Table 2. Number of fragments (Frag), reads (Read) and sequenced bases (bps) in each design for n DNA fragments.

Design	Frag	Read	bps
SE36	n	n	36n
SE50	n	n	50n
SE75	n	n	75n
SE101	n	n	101n
PE36	n	2n	72n
PE50	n	2n	100n
PE75	n	2n	150n
PE101	n	2n	202n

Supplementary Table 3. Abbreviations for the designs of interest.

Read length	36	50	75	101
PE	PE36	PE50	PE75	PE101
SE	SE36	SE50	SE75	SE101

Supplementary Table 4. Abbreviations of Bowtie alignment strategies.

Abbreviations	Command
v0 or BOWTIEv0	Bowtie -v 0
v1 or BOWTIEv1	Bowtie -v 1
v2 or BOWTIEv2	Bowtie -v 2
v3 or BOWTIEv3	Bowtie -v 3

Abbreviations of Bowtie alignment strategies. Prefix "BOWTIE" is also occasionally ignored to save space.

Supplementary Table 5. Abbreviations of BWA alignment strategies.

BWA command	Uni	UR	All
BWA -q 0 -o 0 -n 0.04	BWAq0o0Uni	BWAq0o0UR	BWAq0o0
BWA -q 20 -o 0 -n 0.04	BWAq20o0Uni	BWAq20o0UR	BWAq20o0
BWA -q 20 -o 1 -n 0.04	BWAq20o1Uni	BWAq20o1UR	BWAq20o1
BWA -q 20 -o 1 -n 8			BWAq20o1n8

Abbreviations of BWA alignment strategies. "Uni" stands for the post-processing strategy on BWA output which only keeps the uniquely mapped reads (or read pairs for PE). "UR" represents another such strategy which also keeps the read pairs with one uniquely mapped end for PE, and is identical to Uni for SE.

Supplementary Table 6. Abbreviations for the design-Bowite alignment combinations.

	PE36	PE50	PE75	PE101	SE36	SE50	SE75	SE101
BOWTIEv1	PE36v1	PE50v1	PE75v1	PE101v1	SE36v1	SE50v1	SE75v1	SE101v1
BOWTIEv2	PE36v2	PE50v2	PE75v2	PE101v2	SE36v2	SE50v2	SE75v2	SE101v2
BOWTIEv3	PE36v3	PE50v3	PE75v3	PE101v3	SE36v3	SE50v3	SE75v3	SE101v3

Supplementary Table 7. Abbreviations for PE designs with half of the fragments.

PEhalf	a random sample of half of the pairs from the corresponding PE design
PE36v1half	a random sample of half of the aligned pairs from PE36v1

Supplementary Table 8. The read error distribution used in simulation.

True letter Assigned letter	A	C	G	T	N
A	0	0.3	0.3	0.3	0.1
C	0.3	0	0.3	0.3	0.1
G	0.3	0.3	0	0.3	0.1
T	0.3	0.3	0.3	0	0.1
N	0.25	0.25	0.25	0.25	0

Since this is a conditional distribution given that there is an error, the diagonal entries are 0.

Effect of the design on the alignment

Supplementary Table 9. Percentages of aligned reads for replicate 1 of CTCF.

Design	v0	v1	v2	v3	q20o1Uni	q20o1UR	q20o1
SE36	72.68	85.51	88.69	89.59	88.99	88.99	97.54
SE50	69.41	84.75	89.46	91.18	91.57	91.57	97.32
SE75	61.61	79.65	86.81	90.13	93.15	93.15	96.79
SE101	54.37	73.27	81.85	86.40	93.49	93.49	96.40
PE36	63.40	83.62	88.95	90.32	82.99	93.93	97.70
PE50	57.59	79.88	87.28	90.06	87.09	95.14	97.91
PE75	47.46	71.05	81.08	86.03	89.64	95.87	97.88
PE101	39.15	62.33	73.51	79.77	90.13	96.11	97.76

Supplementary Table 10. Percentages of aligned reads for replicate 2 of CTCF.

Design	v0	v1	v2	v3	q20o1Uni	q20o1UR	q20o1
SE36	83.23	87.96	88.50	88.72	88.76	88.76	98.45
SE50	83.17	89.90	90.84	91.22	91.79	91.79	98.27
SE75	80.09	89.66	91.37	92.04	93.94	93.94	97.97
SE101	71.77	85.47	88.82	90.21	94.55	94.55	97.78
PE36	80.59	88.98	89.40	89.36	82.50	93.46	97.66
PE50	77.23	88.89	90.17	90.53	87.41	94.74	97.79
PE75	69.81	85.66	88.33	89.27	90.99	95.69	97.90
PE101	57.00	78.04	83.31	85.46	91.93	96.11	97.93

Supplementary Table 11. Percentages of aligned reads for replicate 1 of MAFK.

Design	v0	v1	v2	v3	q20o1Uni	q20o1UR	q20o1
SE36	76.76	81.95	82.68	83.00	82.90	82.90	97.49
SE50	78.39	85.30	86.37	86.84	87.24	87.24	96.90
SE75	77.46	86.82	88.36	88.96	90.34	90.34	95.95
SE101	74.45	85.86	87.96	88.72	91.20	91.20	95.35
PE36	75.27	84.4	84.68	84.40	75.45	88.79	95.84
PE50	73.41	85.48	86.56	86.74	81.87	91.21	95.91
PE75	68.39	84.19	86.32	86.85	86.80	92.75	95.77
PE101	63.05	81.55	84.76	85.66	88.28	93.12	95.45

This is a replicate of Table 2.

Supplementary Table 12. Percentages of aligned reads for replicate 2 of MAFK.

Design	v0	v1	v2	v3	q20o1Uni	q20o1UR	q20o1
SE36	77.18	82.05	82.78	83.10	82.99	82.99	97.41
SE50	78.95	85.40	86.42	86.91	87.30	87.30	96.85
SE75	78.02	86.88	88.37	89	90.37	90.37	95.95.00
SE101	74.93	85.91	87.97	88.76	91.25	91.25	95.4
PE36	75.98	84.53	84.86	84.66	75.45	88.98	95.78
PE50	74.40	85.63	86.67	86.90	81.91	91.24	95.82
PE75	69.36	84.30	86.36	86.94	86.84	92.70	95.68
PE101	63.73	81.62	84.75	85.69	88.30	93.14	95.43

Supplementary Table 13. Percentages of aligned reads for replicate 1 of BHLHE40.

Design	v0	v1	v2	v3	q20o1Uni	q20o1UR	q20o1
SE36	77.84	83.00	83.71	83.99	83.93	83.93	97.73
SE50	79.31	86.28	87.34	87.78	88.20	88.20	97.25
SE75	77.79	87.55	89.22	89.83	91.22	91.22	96.43
SE101	74.39	86.42	88.75	89.57	92.06	92.06	95.89
PE36	76.29	85.59	85.90	85.55	77.02	89.63	96.47
PE50	74.12	86.48	87.71	87.88	83.27	92.04	96.54
PE75	68.14	84.69	87.17	87.83	88.01	93.57	96.46
PE101	63.08	81.86	85.47	86.52	89.42	93.86	96.15

Supplementary Table 14. Percentages of aligned reads for replicate 2 of BHLHE40.

Design	v0	v1	v2	v3	q20o1Uni	q20o1UR	q20o1
SE36	77.48	82.69	83.44	83.75	83.66	83.66	96.71
SE50	78.73	85.72	86.82	87.28	87.69	87.69	96.22
SE75	77.01	86.75	88.45	89.10	90.50	90.50	95.39
SE101	73.43	85.43	87.80	88.67	91.21	91.21	94.81
PE36	75.60	84.93	85.36	85.09	76.96	89.10	95.48
PE50	73.32	85.67	86.97	87.22	82.90	91.36	95.55
PE75	67.26	83.74	86.28	86.99	87.35	92.74	95.43
PE101	61.91	80.71	84.40	85.53	88.59	92.96	95.08

Supplementary Table 15. Percentage of aligned simulated reads.

Design	v0	v1	v2	v3	q0o0Uni	q20o0Uni	q20o1Uni	q0o0UR	q20o0UR	q20o1UR	q0o0	q20o0	q20o1
SE36	78.46	83.63	84.23	84.41	84.23	84.27	84.42	84.23	84.27	84.42	98.52	98.58	98.75
SE50	81.20	88.99	90.15	90.51	90.46	91.13	91.46	90.46	91.13	91.46	97.55	98.36	98.70
SE75	77.68	89.57	91.97	92.77	93.12	95.76	96.46	93.12	95.76	96.46	94.99	97.88	98.59
SE101	65.77	79.85	84.02	86.1	88.41	96.42	97.48	88.41	96.42	97.48	89.12	97.44	98.53
PE36	78.34	89.83	90.26	89.69	73.16	73.22	73.49	97.33	98.68	98.83	98.67	98.69	98.75
PE50	75.00	90.19	92.18	92.49	83.00	84.24	84.82	94.78	94.79	94.85	98.7	98.88	98.98
PE75	63.00	83.19	87.52	88.92	87.42	92.41	93.77	97.34	97.48	97.57	98.7	99.03	99.12
PE101	44.89	64.98	71.77	75.28	78.83	93.66	95.67	98.31	98.6	98.68	97.56	98.97	99.12

Median alignment percentages of reads across 5 simulation replicates.

Supplementary Table 16. Percentage of correctly aligned simulated reads.

Design	v0	v1	v2	v3	q0o0Uni	q20o0Uni	q20o1Uni	q0o0UR	q20o0UR	q20o1UR	q0o0	q20o0	q20o1
SE36	99.22	99.2	99.18	99.17	99.18	99.18	99.18	99.18	99.18	99.18	87.05	87.04	87.03
SE50	99.28	99.25	99.23	99.22	99.21	99.21	99.2	99.21	99.21	99.2	93.58	93.48	93.52
SE75	99.35	99.34	99.33	99.32	99.31	99.29	99.29	99.31	99.29	99.29	97.95	97.78	97.79
SE101	99.38	99.37	99.37	99.36	99.35	99.32	99.32	99.35	99.32	99.32	98.83	98.6	98.61
PE36	99.93	99.86	99.8	99.79	100	100	100	99.99	99.99	99.99	99.16	99.16	99.15
PE50	99.98	99.98	99.96	99.96	100	100	100	99.96	99.96	99.96	99.63	99.62	99.61
PE75	100	100	99.99	99.99	100	100	100	99.98	99.98	99.98	99.8	99.79	99.79
PE101	100	100	100	100	100	100	100	99.99	99.99	99.99	99.87	99.84	99.84

Median correct alignment percentages over 5 simulation replicates.

Effect of design on peak calling

Supplementary Table 17. CTCF peak list differences between PE and SE, and between long and short reads.

PE36v1/SE36v1	10000	20000	30000	40000	50000	60000	70000	80000	90000
rank≤ M	9699	19639	29437	38712	47810	57028	66260	75354	84484
M <rank≤ $1.2M$	216	326	509	1171	2009	2656	3199	3831	4204
$1.2M$ <rank≤ 3×10^5	84	32	48	105	166	291	507	767	1237
rank> 3×10^5	1	3	6	12	15	25	34	48	75
SE36v1/PE36v1	10000	20000	30000	40000	50000	60000	70000	80000	90000
rank≤ M	9697	19649	29677	39699	49587	59334	68931	78368	87614
M <rank≤ $1.2M$	259	284	234	168	225	376	690	1055	1462
$1.2M$ <rank≤ 3×10^5	14	10	12	27	28	73	109	226	456
rank> 3×10^5	30	57	77	106	160	217	270	351	468
SE75v2/SE36v1	10000	20000	30000	40000	50000	60000	70000	80000	90000
rank≤ M	9599	19503	29444	39328	49115	58927	68664	78346	87920
M <rank≤ $1.2M$	208	332	365	407	554	709	850	1045	1299
$1.2M$ <rank≤ 3×10^5	179	141	150	194	236	251	341	431	562
rank> 3×10^5	14	24	41	71	95	113	145	178	219
SE36v1/SE75v2	10000	20000	30000	40000	50000	60000	70000	80000	90000
rank≤ M	9603	19492	29388	39198	48906	58600	68283	77931	87422
M <rank≤ $1.2M$	379	501	605	786	1064	1358	1666	1985	2435
$1.2M$ <rank≤ 3×10^5	17	3	0	6	12	20	25	48	96
rank> 3×10^5	1	4	7	10	18	22	26	36	47
PE36v1/SE75v2	10000	20000	30000	40000	50000	60000	70000	80000	90000
rank≤ M	9719	19612	29289	38415	47414	56569	65771	74919	83920
M <rank≤ $1.2M$	271	382	696	1545	2520	3239	3873	4410	4888
$1.2M$ <rank≤ 3×10^5	8	4	13	37	61	182	343	649	1152
rank> 3×10^5	2	2	2	3	5	10	13	22	40
SE75v2/PE36v1	10000	20000	30000	40000	50000	60000	70000	80000	90000
rank≤ M	9715	19634	29587	39534	49402	59212	68837	78366	87554
M <rank≤ $1.2M$	176	206	193	167	212	312	557	824	1285
$1.2M$ <rank≤ 3×10^5	68	88	116	141	166	194	240	349	568
rank> 3×10^5	41	72	104	158	220	282	366	461	593

Supplementary Table 18. BHLHE40 peak list differences between PE and SE, and between long and short reads.

PE36v1/SE36v1	3000	6000	9000	12000	15000	18000	21000	24000	27000
rank$\leq M$	2817	5471	7969	10338	12685	14935	17111	19282	21224
$M <$rank$\leq 1.2M$	174	476	894	1383	1790	2195	2512	2646	2891
$1.2M <$rank$\leq 3 \times 10^5$	8	43	118	254	463	749	1160	1688	2259
rank$> 3 \times 10^5$	1	10	19	25	62	121	217	384	626
SE36v1/PE36v1	3000	6000	9000	12000	15000	18000	21000	24000	27000
rank$\leq M$	2821	5492	8018	10424	12824	15134	17379	19614	21625
$M <$rank$\leq 1.2M$	85	179	279	411	552	727	890	982	1231
$1.2M <$rank$\leq 3 \times 10^5$	7	21	37	69	108	147	271	482	757
rank$> 3 \times 10^5$	87	308	666	1096	1516	1992	2460	2922	3387
SE75v2/SE36v1	3000	6000	9000	12000	15000	18000	21000	24000	27000
rank$\leq M$	2866	5710	8510	11281	14087	16769	19514	22182	24782
$M <$rank$\leq 1.2M$	85	175	295	442	567	743	843	952	1119
$1.2M <$rank$\leq 3 \times 10^5$	32	61	104	137	153	235	321	456	585
rank$> 3 \times 10^5$	17	54	91	140	193	253	322	410	514
SE36v1/SE75v2	3000	6000	9000	12000	15000	18000	21000	24000	27000
rank$\leq M$	2864	5719	8512	11292	14105	16787	19533	22185	24788
$M <$rank$\leq 1.2M$	127	246	399	566	681	930	1094	1341	1627
$1.2M <$rank$\leq 3 \times 10^5$	0	1	10	10	25	44	76	135	199
rank$> 3 \times 10^5$	9	34	79	132	189	239	297	339	386
PE36v1/SE75v2	3000	6000	9000	12000	15000	18000	21000	24000	27000
rank$\leq M$	2797	5454	7943	10328	12668	14929	17167	19323	21266
$M <$rank$\leq 1.2M$	202	519	974	1429	1877	2304	2617	2765	2993
$1.2M <$rank$\leq 3 \times 10^5$	1	21	70	220	411	675	1032	1584	2204
rank$> 3 \times 10^5$	0	6	13	23	44	92	184	328	537
SE75v2/PE36v1	3000	6000	9000	12000	15000	18000	21000	24000	27000
rank$\leq M$	2804	5470	7988	10400	12781	15114	17417	19646	21647
$M <$rank$\leq 1.2M$	81	172	270	405	535	715	854	974	1179
$1.2M <$rank$\leq 3 \times 10^5$	17	29	54	75	127	181	290	457	753
rank$> 3 \times 10^5$	98	329	688	1120	1557	1990	2439	2923	3421

Supplementary Table 19. MAFK peak list differences between PE and SE, and between long and short reads.

PE36v1/SE36v1	1500	3000	4500	6000	7500	9000	10500	12000	13500
rank$\leq M$	1462	2898	4308	5675	6957	8130	9273	10320	11310
$M <$rank$\leq 1.2M$	36	96	174	294	465	694	836	974	1123
$1.2M <$rank$\leq 3\times10^5$	1	4	14	26	70	164	365	650	958
rank$> 3\times10^5$	1	2	4	5	8	12	26	56	109
SE36v1/PE36v1	1500	3000	4500	6000	7500	9000	10500	12000	13500
rank$\leq M$	1462	2900	4310	5680	6966	8155	9309	10372	11371
$M <$rank$\leq 1.2M$	37	99	175	282	406	623	793	933	1073
$1.2M <$rank$\leq 3\times10^5$	1	0	9	22	90	161	309	573	899
rank$> 3\times10^5$	0	1	6	16	38	61	89	122	157
SE75v2/SE36v1	1500	3000	4500	6000	7500	9000	10500	12000	13500
rank$\leq M$	1448	2905	4343	5786	7191	8564	9915	11230	12508
$M <$rank$\leq 1.2M$	43	78	117	157	204	272	316	382	465
$1.2M <$rank$\leq 3\times10^5$	8	16	36	50	87	131	210	297	403
rank$> 3\times10^5$	1	1	4	7	18	33	59	91	124
SE36v1/SE75v2	1500	3000	4500	6000	7500	9000	10500	12000	13500
rank$\leq M$	1448	2902	4340	5782	7189	8564	9907	11217	12492
$M <$rank$\leq 1.2M$	52	97	158	209	285	399	534	699	882
$1.2M <$rank$\leq 3\times10^5$	0	0	1	4	8	15	34	55	89
rank$> 3\times10^5$	0	1	1	5	18	22	25	29	37
PE36v1/SE75v2	1500	3000	4500	6000	7500	9000	10500	12000	13500
rank$\leq M$	1464	2905	4310	5673	6963	8130	9296	10366	11318
$M <$rank$\leq 1.2M$	36	94	186	316	478	719	853	986	1178
$1.2M <$rank$\leq 3\times10^5$	0	0	1	6	49	136	322	602	919
rank$> 3\times10^5$	0	1	3	5	10	15	29	46	85
SE75v2/PE36v1	1500	3000	4500	6000	7500	9000	10500	12000	13500
rank$\leq M$	1464	2910	4315	5681	6974	8158	9343	10430	11392
$M <$rank$\leq 1.2M$	32	75	144	253	379	582	700	841	1004
$1.2M <$rank$\leq 3\times10^5$	4	15	33	47	107	186	345	565	889
rank$> 3\times10^5$	0	0	8	19	40	74	112	164	215

Supplementary Table 20. Numbers of design specific peaks with at least 1.5 fold change in coverage in at least one replicate of the ChIP or the control data.

	CTCF	BHLHE40	MAFK
PE36v1/SE36v1	30 (46)	40 (191)	13 (37)
SE36v1/PE36v1	13 (280)	85 (2099)	4 (33)
SE75v2/SE36v1	33 (139)	69 (252)	9 (28)
SE36v1/SE75v2	5 (24)	7 (227)	3 (16)
PE36v1/SE75v2	11 (22)	15 (156)	7 (34)
SE75v2/PE36v1	31 (374)	126 (2137)	7 (49)

Numbers of design specific peaks with at least 1.5 fold change in coverage in at least one replicate of the ChIP or the control data. Numbers of all design specific peaks are provided in parentheses.

Supplementary Table 21. Design specific peaks of CTCF with at least 1.5 fold change in coverage in at least one replicate of the ChIP or the control data.

	Size	Motif	DNA	LINE	SINE	LTR	SDR
PE36v1/SE36v1	30	1	3	3	9	1	28
SE36v1/PE36v1	13	0	0	2	5	2	2
SE75v2/SE36v1	33	0	2	3	5	6	31
SE36v1/SE75v2	5	0	0	0	1	0	5
PE36v1/SE75v2	11	0	1	3	4	0	10
SE75v2/PE36v1	31	1	2	1	5	3	22

CTCF design specific peaks with at least 1.5 fold change in coverage in at least one replicate of the ChIP or the control data. The column "Size" represents the number of design-specific peaks, and the rest of the columns represent the numbers of peaks with a motif (column 3) or overlapping with different classes of repetitive elements (columns 4-8).

Supplementary Table 22. Design specific peaks of BHLHE40 with at least 1.5 fold change in coverage in at least one replicate of the ChIP or the control data.

	Size	MOTIF	DNA	LINE	SINE	LTR	SDR
PE36v1/SE36v1	40	2	0	1	24	5	10
SE36v1/PE36v1	85	1	4	5	37	8	13
SE75v2/SE36v1	69	0	0	2	42	8	21
SE36v1/SE75v2	7	0	0	1	3	1	3
PE36v1/SE75v2	15	1	0	3	3	0	5
SE75v2/PE36v1	126	25	1	11	37	8	52

Design specific peaks of BHLHE40 with at least 1.5 fold change in coverage in at least one replicate of the ChIP or the control data. The column "Size" represents the number of design-specific peaks, and the rest of the columns represents the the numbers of peaks with a motif (column 3) or overlapping with different classes of repetitive elements (columns 4-8).

Supplementary Table 23. Design specific peaks of MAFK with at least 1.5 fold change in coverage in at least one replicate of the ChIP or the control data.

	Size	Motif	DNA	LINE	SINE	LTR	SDR
PE36v1/SE36v1	13	2	0	3	2	4	6
SE36v1/PE36v1	4	0	0	2	0	0	0
SE75v2/SE36v1	9	1	0	1	2	1	6
SE36v1/SE75v2	3	0	0	1	0	0	0
PE36v1/SE75v2	7	0	0	0	2	0	6
SE75v2/PE36v1	7	1	0	2	0	2	4

Design specific peaks of MAFK with at least 1.5 fold change in coverage in at least one replicate of the ChIP or the control data. The column "Size" represents the number of design-specific peaks, and the rest of the columns represents the the numbers of peaks with a motif (column 3) or overlapping with different classes of repetitive elements (columns 4-8).

Effect of design on ASB detection

Supplementary Table 24. Summary statistics of the overall read counts at ASB loci of CTCF.

Read type	ASB list	Min	1st Quartile	Median	Mean	3rd Quartile	Max
PE	all PE	8	25	52	91.76	115	1477
SE	all PE	5	25	52	96.33	119.8	1519
PE	all SE	5	24	52	91.74	117	1477
SE	all SE	8	25	54	97.76	124.8	1519
PE	PE-specific	8	22.25	49	91.89	117.8	827
SE	PE-specific	5	22	46	94.57	116.8	797
PE	SE-specific	5	19	46	91.86	131	669
SE	SE-specific	8	22	50	98.82	128.8	673

PE and SE read counts are tabulated at the ASB loci detected by AlleleSeq under PE50v1 or SE101v2 of CTCF dataset. The columns are: Read Type: PE or SE; ASB list: indicates the list of ASB detected, where the first four rows are for all ASB detected under PE50v1 (all PE) and under SE101v2 (all SE), and the last four rows are for those that are detected under PE50v1 but not under SE101v2 (PE-specific) and the vice versa (SE-specific).

Supplementary Table 25. Numbers of detected ASB loci overlapping a CTCF motif in both alleles, neither allele, and only the winning allele, and only the losing allele.

PE36v1 vs. SE75v2	neither	win	lose	both
PE36v1 not SE75v2	622	11	8	19
SE75v2 not PE36v1	488	16	1	27
PE36v1 and SE75v2	798	93	7	175

PE36v1 vs. SE36v1	neither	win	lose	both
PE36v1 not SE36v1	817	36	12	55
SE36v1 not PE36v1	205	2	1	3
PE36v1 and SE36v1	603	68	3	139

PE75v2 vs. SE75v2	neither	win	lose	both
PE75v2 not SE75v2	1128	17	8	46
SE75v2 not PE75v2	164	0	1	3
PE75v2 and SE75v2	1122	109	7	199

PE75v2 vs. PE36v1	neither	win	lose	both
PE75v2 not PE36v1	1162	25	5	56
PE36v1 not PE75v2	332	3	5	5
PE75v2 and PE36v1	1088	101	10	189

SE75v2 vs. SE36v1	neither	win	lose	both
SE75v2 not SE36v1	713	41	4	65
SE36v1 not SE75v2	235	2	0	5
SE75v2 and SE36v1	573	68	4	137

PE50v1 vs. SE101v2	neither	win	lose	both
PE50v1 not SE101v2	699	12	2	17
SE101v2 not PE50v1	606	7	1	24
PE50v1 and SE101v2	1042	110	10	194

PE50v1 vs. SE50v1	neither	win	lose	both
PE50v1 not SE50v1	951	35	8	39
SE50v1 not PE50v1	190	0	1	5
PE50v1 and SE50v1	790	87	4	172

PE101v2 vs. SE101v2	neither	win	lose	both
PE101v2 not SE101v2	1152	23	10	32
SE101v2 not PE101v2	228	0	1	2
PE101v2 and SE101v2	1420	117	10	216

PE101v2 vs. PE50v1	neither	win	lose	both
PE101v2 not PE50v1	1197	20	9	39
PE50v1 not PE101v2	366	2	1	2
PE101v2 and PE50v1	1375	120	11	209

SE101v2 vs. SE50v1	neither	win	lose	both
SE101v2 not SE50v1	900	30	7	49
SE50v1 not SE101v2	232	0	1	8
SE101v2 and SE50v1	748	87	4	169

The detected ASB events were classified into three groups as common (e.g., PE36v1 and SE75v2) and set differences (e.g., PE36v1 not SE75v2 and SE75v2 not PE36v1) for each pair of designs of the CTCF dataset. The winning alleles are the same for those detected by both designs. CTCF motifs that overlap these SNPs in either allele were identified using FIMO in the default setting. The number of detected ASB events overlapping a motif in both alleles (both), only the winning allele (win), only the losing allele (lose) and in neither allele (neither) are reported for each pair of designs.

Tables for Supplementary notes

Supplementary Table 26. Number of non-overlapping intervals that are enriched of false alignments.

Design	v0	v1	v2	v3	q0o0Uni	q20o0Uni	q20o1Uni	q0o0UR	q20o0UR	q20o1UR	q0o0	q20o0	q20o1
SE36	1	1	1	1	1	1	1	1	1	1	82	73	73
SE50	2	2	2	2	2	2	2	2	2	2	50	54	52
SE75	1	2	2	2	2	2	2	2	2	2	31	35	32
SE101	0	0	0	0	0	0	0	0	0	0	18	23	24
PE36	0	0	0	0	0	0	0	0	0	0	42	45	52
PE50	0	0	0	0	0	0	0	0	0	0	23	24	26
PE75	0	0	0	0	0	0	0	0	0	0	11	12	13
PE101	0	0	0	0	0	0	0	0	0	0	7	10	8

Median number of non-overlapping intervals (200 bps long) that are enriched of false alignments over 5 replicates of simulated CTCF reads.
